# Precarious Employment and Stress: The Biomedical Embodiment of Social Factors. PRESSED Project Study Protocol

**DOI:** 10.3389/fpubh.2021.649447

**Published:** 2021-03-30

**Authors:** Mireia Bolibar, Francesc Xavier Belvis, Pere Jódar, Alejandra Vives, Fabrizio Méndez, Xavier Bartoll-Roca, Oscar J. Pozo, Alex Gomez-Gomez, Eva Padrosa, Joan Benach, Mireia Julià

**Affiliations:** ^1^Department of Sociology, University of Barcelona, Barcelona, Spain; ^2^Research Group on Health Inequalities, Environment, and Employment Conditions (GREDS-EMCONET), Department of Political and Social Sciences, Universitat Pompeu Fabra, Barcelona, Spain; ^3^Johns Hopkins University – Universitat Pompeu Fabra Public Policy Center, Barcelona, Spain; ^4^Department of Public Health, CEDEUS, Pontificia Universidad Católica de Chile, Santiago, Chile; ^5^Barcelona Public Health Agency, Barcelona, Spain; ^6^Integrative Pharmacology and Systems Neuroscience Group, IMIM (Hospital del Mar Medical Research Institute), Barcelona, Spain; ^7^Department of Experimental and Health Sciences, Universitat Pompeu Fabra, Barcelona, Spain; ^8^Transdisciplinary Research Group on Socioecological Transitions (GinTRANS2), Universidad Autónoma de Madrid, Madrid, Spain

**Keywords:** precarious employment, stress, health inequalities, stress biomarkers, social support networks, in-work poverty, insecurity, psychosocial risks

## Abstract

The PRESSED project aims to explain the links between a multidimensional measure of precarious employment and stress and health. Studies on social epidemiology have found a clear positive association between precarious employment and health, but the pathways and mechanisms to explain such a relationship are not well-understood. This project aims to fill this gap from an interdisciplinary perspective, integrating the social and biomedical standpoints to comprehensively address the complex web of consequences of precarious employment and its effects on workers' stress, health and well-being, including health inequalities. The project objectives are: (1) to analyze the association between multidimensional precarious employment and chronic stress among salaried workers in Barcelona, measured both subjectively and using biological indicators; (2) to improve our understanding of the pathways and mechanisms linking precarious employment with stress, health and well-being; and (3) to analyze health inequalities by gender, social class and place of origin for the first two objectives. The study follows a sequential mixed design. First, secondary data from the 2017 Survey on Workers and the Unemployed of Barcelona is analyzed (*N* = 1,264), yielding a social map of precarious employment in Barcelona that allows the contextualization of the scope and characteristics of this phenomenon. Drawing on these results, a second survey on a smaller sample (*N* = 255) on precarious employment, social precariousness and stress is envisaged. This study population is also asked to provide a hair sample to have their levels of cortisol and its related components, biomarkers of chronic stress, analyzed. Third, a sub-sample of the latter survey (n = 25) is selected to perform qualitative semi-structured interviews. This allows going into greater depth into how and why the experience of uncertainty, the precarization of living conditions, and the degradation of working conditions go hand-in-hand with precarious employment and have an impact on stress, as well as to explore the potential role of social support networks in mitigating these effects.

## Introduction

### Employment Conditions as an Emerging Social Determinant of Health

The last quarter of the twentieth century saw the emergence of a paradigm shift in understanding and explaining health and health inequalities: the perspective of *social determinants of health*. This approach emphasizes the conditions in which people are born, live, work and age, including health systems (1); conditions (and relations) understood as being the result of the unequal distribution of money, power, and resources according to the social structure. Therefore, this perspective highlights the need to consider socioeconomic inequalities to understand health inequalities and the need to study the mechanisms whereby the social structure is embodied (i.e., the process of “disease embodiment”).

*Employment* is considered one of the most relevant social determinants of health. Given the central role of employment in the organization of the social structure and in the definition of people's living conditions, its characteristics are key to explaining the health and welfare of workers, families and communities (2). From this perspective of the social determinants of health, the effects of employment on health and well-being thus go beyond a limited perspective on psychosocial, physical, chemical and environmental risk factors that occur in the workplace, which focus on people's immediate/proximal environment. It rather understands employment as a historical phenomenon linked to power relations between employers and workers, and to the particular forms of labor market regulation and the welfare state.

In this regard, the expansion of neoliberal policies and changes in labor markets that took place in the late twentieth century, which led to the erosion of the “standard employment relationship” and to the proliferation of flexible (sometimes called “non-standard”) forms of employment (3), acquire special relevance. Indeed, they led to profound changes in employment conditions and relations through modifying collective agreements, limiting social security benefits or deregulating contractual relations (4). The Great Recession of 2008 exacerbated these trends yet further. The economic crisis and fiscal austerity policies eroded levels of social and labor protection, and the application of social and labor reforms brought about massive layoffs and a further decrease in the protection of employment, leading to widespread unstable unemployment (5, 6) and increased precarity of employment conditions. In this scenario, precarious employment should take center stage in the agenda aimed at identifying and combating emerging contemporary threats to the health of the population, and to the generation of health inequalities.

### Precarious Employment and Health: Existing Evidence and Research Challenges

#### Precarious Employment: Definition and Operationalization

Precarious employment is a general term that refers to insecure employment conditions, implying a deficit in some of the dimensions of “employment quality” (7). The term “precarious” was coined by French sociologists in the early 1980s to describe temporary or seasonal work (8). Nevertheless, there is still no consensus in the scientific literature regarding the definition of precarious employment.

Social scientists often stress the importance of single aspects such as insecurity, flexibility, chronic uncertainty, and vulnerability. For years, many authors analyzed precarious employment using one-dimensional approaches such as the temporary nature of the contract or the self-perception of job insecurity. However, precarious employment is a widespread phenomenon that affects both temporary and permanent workers and, therefore, heterogeneity between and within the different types of contract warrants using a multidimensional construct of precarious employment, not solely the analysis of the type of contract as a one-dimensional indicator (9). Accordingly, in recent decades multidimensional approaches have emerged, which address a broader view of the construct (10).

Following this logic, for two decades, the GREDS-EMCONET research group has made substantial efforts to operationalize the concept of precarious employment by developing the Employment Precariousness Scale (EPRES). It is a multidimensional construct that has been empirically validated in several countries such as Spain, Chile and Sweden (11–15), and is currently being tested in Belgium and Finland. EPRES is based on the conceptual work by Rodgers, who considered four dimensions of precarious employment (temporariness, lack of protection -individualized negotiation between employees and employers-, vulnerability, and wages) (16) and two further dimensions based on a qualitative study conducted in Spain (17) that pointed out the importance of the scope of rights to which workers are entitled and of their ability to exercise them in the workplace. As a result, EPRES has a total of 22 items divided into six dimensions: temporariness (contract duration and tenure), disempowerment (level of negotiation of employment conditions), vulnerability (defenselessness to authoritarian treatment), wages (low or insufficient; possible economic deprivation), rights (entitlement to workplace rights and social security benefits), and exercise of rights (powerlessness, in practice, to exercise workplace rights) (11, 18, 19).

EPRES has proved to be capable of capturing concisely the complexity of the forms of employment that define precarious employment and has therefore allowed studying its prevalence and its association with health among the working population. For example, using data from the EPRES for Spain, the prevalence of precarious employment among salaried workers in 2005 was 48%, and 50% in 2010. Therefore, in this project we will use this construct to measure precarious employment.

#### The Relations Between Precarious Employment and Health

The various research approaches to precarious employment and health in flexible labor markets are a great but heterogeneous source of information. Research in social epidemiology has addressed this issue from three main angles, showing the harmful health effects of: (a) temporary employment; (b) perceived employment insecurity and staff restructuring and downsizing; and (c) multi-dimensional approaches to precarious employment (10, 20–22).

Instability or temporary employment is one of the most commonly used one-dimensional indicators due to its widespread availability. Although studies have generally shown a positive association between temporary employment and psychological morbidity, there is still heterogeneity in this field of research. Some authors find that the mental and self-perceived health of temporary workers is worse than that of permanent ones (23), whereas others find no differences (24), and still others find that it is even better (25). These disparities might be due to the heterogeneity among temporary workers, which reinforces the need for applying multidimensional approaches. Moreover, a recent study shows that although the prevalence of precarious employment is higher among temporary than permanent workers, the association with poor mental health is greater among workers with permanent contracts than those with temporary contracts, suggesting that precarious employment might be more harmful for permanent workers (9).

With regard to perceived employment insecurity, the results are more homogeneous and show a positive association with various health outcomes (26, 27). Notwithstanding, this indicator is based on perceptions, which may be influenced by external factors such as economic crises or staff downsizing.

The relationship between precarious employment and health using multidimensional measures is still limited but has increased in recent years, proving to be an insightful tool to comprehensively address the relationship between precarious employment and health (28). In Canada, using an eight-dimensional approach, it was shown that some dimensions of precarious employment such as low income, lack of an annual pay rise, no overtime pay and a manual job preceded an increased risk of adverse health outcomes (29). To be more specific, the Canadian researchers identified that both strain brought up by precarity in the employment relationship considered as a whole and the strain resulting from the dimensions considered taken separately were related to several health indicators (30). Subsequently, again in Canada, precarious employment as measured by the Employment Precarity Index was associated with worse mental health outcomes and poorer self-perceived health and household well-being (31). Research using the EPRES multidimensional scale has shown associations between precarious employment and poor mental health in Spain and Catalonia (9, 32, 33), and with poor self-perceived health (9, 32). This relationship was also found in a sample of non-standard employees of Stockholm County (Sweden) (34). Qualitative evidence collected in Spain from Spanish (17) and immigrant workers (35) also confirms this finding.

#### Challenges for Research

Despite the growing evidence of the association between precarious employment and health, social epidemiology studies tend to focus on temporal trends and on patterns according to the social profiles of workers, but the explanatory mechanisms underlying this relationship, both in biomedical and in socioeconomic terms, have not yet been sufficiently studied (10, 36). However, this is essential to design policies intended to improve health and quality of life, especially in respect of the most vulnerable workers, and to fight against health inequalities. This project therefore aims to fill this gap from the two standpoints: the biomedical and the socioeconomic, by means of a mixed-methods design that enables pooling the existing partial evidence on the complex web of consequences of precarious employment and its association with health and well-being. The two following sections deal with these two relevant aspects.

### Precarious Employment and Stress

Stress is currently a major social problem, with considerable and persistent effects at all levels. Several studies show that stressful events are associated with poor physical and mental health through psychophysiological mechanisms (37–39), which may cause many health problems including cardiovascular diseases, metabolic syndrome, osteoporosis, and depression (40). The term “stress” was first coined in the world of medicine in 1956 by Selye (41) to refer to unpleasant environmental events and to the physiological reactions they cause. But it was not until 1967 that psychiatrists Holmes and Rahe conducted population studies to examine the impact of stressful experiences (37). Subsequent definitions focused on the perceived imbalance between demand and the individual's capacity to respond to it (under conditions in which the failure to resolve the situation has important perceived consequences for the individual), whereby two main components of stress are differentiated: its causes or environmental stressors, and its effects, that is, the subjective reaction consisting of an emotional component and one of cognitive perception.

Research analyzing the relationship between precarious employment and stress is scarce. Although some studies have analyzed this relationship using some of the one-dimensional approaches mentioned above, including perceived job insecurity and temporary employment (42, 43), to our knowledge, none has analyzed the links between multidimensional precarious employment and stress. Furthermore, stress in this field of research is usually measured through self-assessed indicators, and the use of more objective measures such as biomarkers is still in its infancy.

To measure stress from an epidemiological point of view, various types of instruments have been developed, depending on the theoretical approach used to address it (44). The first is the environmental approach, which focuses on evaluating the causes of stress or stressors. The second approach is psychological and is based on the individual's subjective evaluation of how they deal with the stressful situation. The last is the biological approach, which consists of measuring the activation of specific physiological systems involved in the response to stress (44). Moreover, to analyse stress, it is also important to note the duration of exposure to the stressor, i.e., whether exposure is acute or chronic.

Epidemiological research has mainly relied on self-reported measures of stress, leaving the biological approach rather unexplored. However, the latter really allows capturing the actual embodiment of stressors in altered biological processes and offers an “objective” measure of stress that is less dependent upon subjective assessments. Within this category, in recent years much attention has been given to the study of cortisol as a biomarker of stress. Cortisol is a glucocorticoid hormone released after stimulation of the hypothalamic-pituitary-adrenal axis (HPA), which is activated by stress and interacts with the immune system and the autonomic nervous system, which are also involved in the body's response to acute and chronic stress. Cortisol can be measured reliably in various biological media. More specifically, blood serum and saliva have been used to measure acute cortisol concentrations and urine for short-term concentrations (45, 46). However, several studies addressing stress in the workplace that used serum or saliva samples yielded highly variable and even conflicting results (47). To a large extent, this is because work-related stress factors are likely to have long-term effects, and the accurate representation of chronic stress levels in these samples is more difficult because of the following: they are subject to the circadian rhythm of cortisol and therefore suffer from poor long-term resolution (48); and, more importantly, they are highly reactive to acute and transient stress factors, obscuring the influence of chronic stress factors (49).

Accordingly, to study *chronic* stress the most suitable means to date is the extraction of cortisol from human hair. This can provide valuable information about exposure to stress in a defined period of time, which can be up to several months (50, 51): since hair grows at a rate of ~1 cm per month, and hormones are transported from blood flow to hair follicles, this method enables a retrospective examination of cortisol levels, allowing the establishment of a basal level and the consideration of stress events that have occurred during the period of examination (i.e., length of hair analyzed) (51). Hair cortisol concentration (HCC) has already been used in occupational health research to study the relationship between one of the main chronic stressors, unemployment, and health. According to this evidence, unemployment has been documented to have an impact on people's health and to be associated with increased cortisol levels (52). It has also been reported that male shift workers under the age of 40 had significantly higher cortisol levels than day workers (53) and that an increase in perceived job insecurity is (slightly) correlated with higher levels of cortisol (54). Nevertheless, the relationship between HCC and different measures of self-reported stress is under discussion (51, 55), and some authors are already delving into cortisol's related compounds in search of better biomarkers of chronic stress (56).

### Social Pathways of Precarious Employment and Health

**Figure 1:**
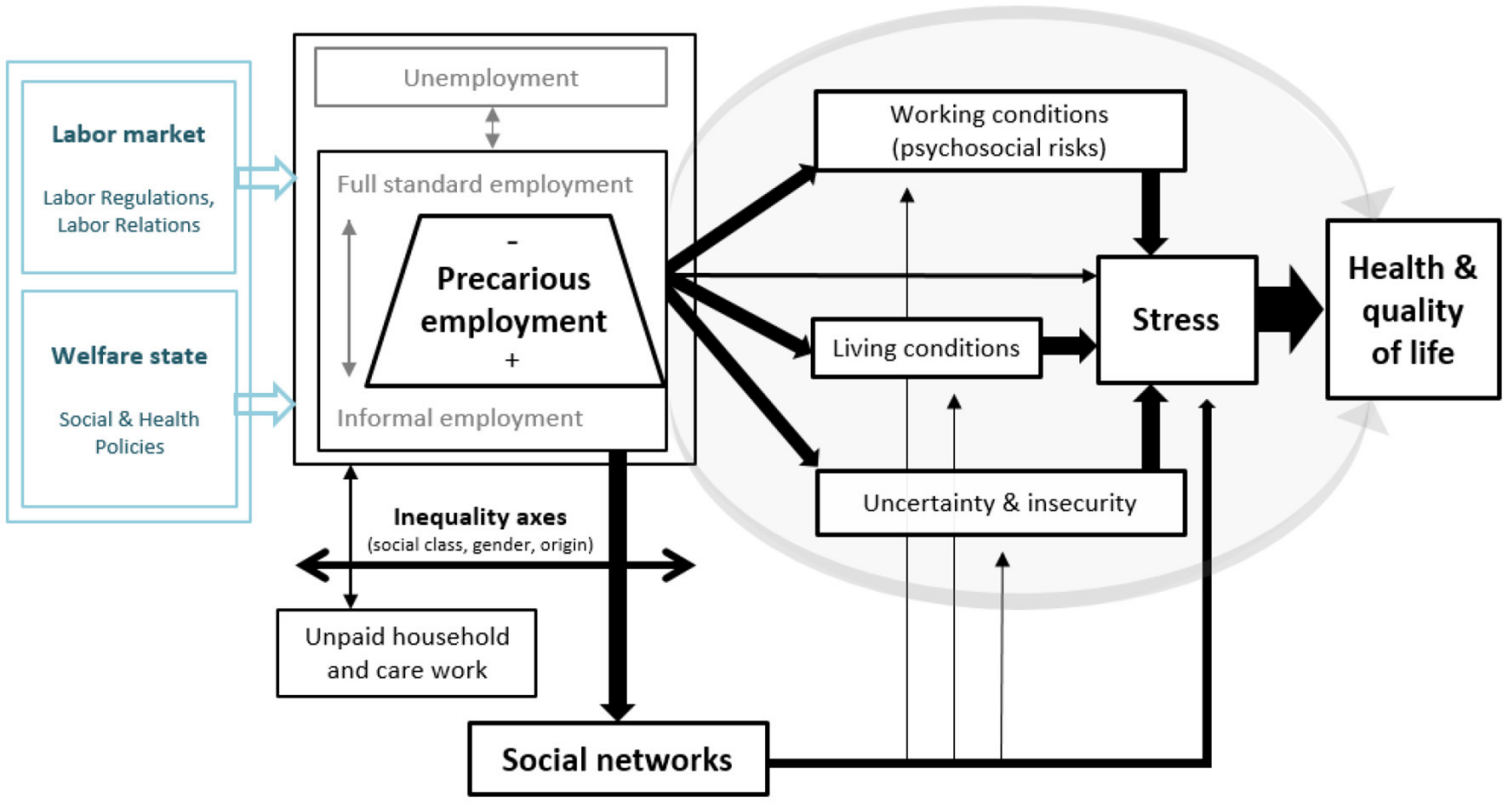
Conceptual framework of precarious employment and the pathways through which it has an impact on stress and health (in black, the core elements addressed empirically in the PRESSED project). Source: Authors' own based on the models of Benach et al. (60) and Julià et al. (19).

The nature and extent of precarious employment has multiple effects on various social dimensions indirectly linked to employment and work (57), triggering a multiple social vulnerabilization process that defines the “precarious condition” (58, 59). In addition to the direct impact that precarious employment has on health, there are four social consequences of precarious employment that might have an impact on workers' health: (i) the exposure to occupational risks factors suffered by precarious workers, (ii) impoverishment and material deprivation, (iii) uncertainty, and (iv) the erosion of social support networks (Figure 1). We consider these as being pathways within which particular mechanisms operate to trigger adverse health outcomes.

The first pathway that potentially explains the relationship between precarious employment and adverse health outcomes involves the greater exposure of precarious workers to poor working conditions, including more adverse psychosocial factors. Psychosocial risks can be defined as work characteristics or conditions that undermine self-efficacy, job control and self-esteem, generating a stressful experience that exposes the individual to the risk of suffering physical and/or mental illnesses (61, 62). Psychosocial risk models put the focus primarily on conditions in the workplace and do not delve into the more general employment conditions as a possible determinant of a stressful psychosocial environment. However, some studies comparing standard and non-standard employment contracts have shown an overall picture of more adverse health-related working conditions in non-standard employment contracts (63–65). Moreover, others also reveal that temporary employment is associated with more passive, high effort types of jobs, with little demand for tasks and less autonomy (66). The literature focusing on Europe suggests that temporary workers are more susceptible to suffering the negative consequences of internal flexibility (e.g., odd working hours, shifts, variable wages, etc.) and from the point of view of work intensification, factors of discomfort, and even physical risks posed by the working conditions (67). Additionally, evidence suggests that these adverse social experiences may be linked to the precarious employment status itself (11).

The other pathways refer to the cascade of consequences of precarious employment beyond the workplace. They might all be considered forms of “social precariousness.” We differentiate them with the aim of distinguishing analytically specific dimensions of such social precariousness related to material living conditions, the subjective experience of insecurity and social network configurations, as three distinct pathways whereby precarious employment might be related to stress, health, and well-being.

Following this logic, we identify a second pathway: adverse living conditions. Precarious employment has major consequences on the material level, creating situations of both absolute and relative deprivation. As the Foessa Foundation points out, the increasing flexibilization of the Spanish labor market and the subsequent increase in precarious employment have emphasized an already stark problem, in-work poverty, as one of the features characterizing households in the post-crisis context (68). Conditions such as underemployment and discontinuity in employment are factors that contribute to the increase in in-work poverty (69). Indeed, since they have lower, more variable and less predictable wages, precarious workers suffer the greatest pressure of wage flexibility and their income is highly insecure (70). Moreover, in most post-industrial societies social protection schemes are tightly linked to employment and its characteristics (5). Accordingly, most precarious workers do not meet the criteria to benefit from these social transfers and key services, including unemployment benefits, pensions, etc., which hinders workers' social protection and the decommodification of their income (70–72). Consequently, they are unprotected from sudden income stoppages, thus being susceptible to material deprivation and debt. That being said, in-work poverty emerges as a potential pathway or mediating mechanism of the relationship between precarious employment and health. It is commonly assumed that the economic condition is one of the most important factors determining people's health, as it directly affects virtually all of the social determinants of health (such as housing, diet, etc.) (73, 74). Harsh material conditions of existence and feelings arising from absolute and relative poverty not only directly harm the physical health, but also lead to exposure to psychosocial stress factors with a major impact on mental health, thus becoming the root of a large number of mental psychological disorders (75, 76). Indeed, stress, in particular, through neuroendocrine mechanisms, is the channel through which poverty has been noted to have a negative impact on mental health (77), but also on certain physical illnesses, especially those related with the cardiovascular system (78).

Thirdly, precarious employment entails a social and subjective experience of vulnerability (79). The uncertainty and insecurity of existence can be considered a constituent element of capitalism, which ensures the need for the working class to sell its labor. However, with the rise of the neoliberal capitalist model, this trend has been exacerbated. The flexibility of the labor market, the financialization of the economy and the weakening of the Welfare State (80, 81) are factors specific to the socioeconomic organization of the last 4 years that have helped erode the social norm of employment (82), thereby questioning the guarantees of social citizenship built on employment (81). Castel (81) describes these guarantees and resources as the basis to which the worker can resort to govern the present and command their future. They provide work with a statute that includes non-market guarantees such as the right to a minimum wage, labor law protections, coverage for accidents and illness, the right to retirement, etc. that protect the worker, i.e., that will allow them to be able to deal with the main risks of life, so that they are not condemned to living day after day in the anguish of tomorrow. Thus, precarious employment gives rise to great vulnerability to social contingencies or risks that compromise individuals' ability to ensure their social independence by themselves, leaving the most precarious population at the mercy of imponderables that can degrade their social status (81). As a result, precarious employment hinders the capacity for agency and the self-perception of control over the future, limiting, and restricting temporary horizon-building (83). Precarious employment spreads uncertainty, unpredictability and the experiential condition of a limited, precarious existence in the present (70, 84, 85), even among those who do not suffer precarious employment, but may perceive it as a threat (84). Through danger, destabilization, fear, and contingency, precarious employment generates modes of subjectivation, creating a generalized, permanent state of insecurity (85). This has a major impact on the structuring of the course of life and its main transitions. Precarious employment hinders making fundamental decisions regarding personal and family life. Short, stable and predictable transitions to and within employment, characteristics of a “Fordist” social structure in which life experiences were relatively standardized and homogeneous, have given way to more protracted, non-linear, increasingly diverse, fragmented, and in some aspects less predictable transitions (86), limiting the capacity for agency in the development of one's own biography or life path.

These experiences of insecurity, lack of control and future perspectives have been identified as powerful social stressors, which in turn can be linked to outcomes of adverse health and well-being (80, 87). Job insecurity, especially, has received particular attention, and has been identified as a major mechanism linking flexible employment with negative psychological well-being and several somatic health outcomes (88).

Finally, in this project we are interested in deepening into the role of social support networks in the relationship between precarious employment and health, particularly stress and well-being. Informal social networks (i.e., interactions between individuals and the resulting relational structures) are a source of economic, material, emotional, and informative support (89–91) and are particularly important among people and households who are in a position of greater vulnerability (68, 92). There is a long tradition of studies that demonstrate the relationship between support networks and health, highlighting the protective role of relational resources and support (93). In addition to their direct effect on health, Cohen and Wills (94) highlighted the role of networks as a buffer, i.e., their ability to protect people from the potentially pathogenic influence of stressful events. Numerous studies prove this role for stressful labor-related situations, such as unemployment (95–98), job seeking (99), intense work (100), or employment insecurity (101).

However, some researchers point out that the extension of precariousness might also lead to the erosion of social support networks (shrinking family- and friends-based networks, community bonds, relationships created within trade unions and associations, etc.) (102, 103). Accordingly, precarious employment has brought about new forms of individualization (85), causing the most precarious workers not to feel part of a caring community (70). In this vein, academic research has shown that characteristics of networks that surround an individual (their “personal network”) reflect an individual's life course (104) as ties are created in sociability spheres in which people engage throughout their lives (105). Hence the experience of precarious employment can leave a mark on the support network that some precarious people have, eroding their capacity for support and thus buffer the cascade of social stressors that result from the experience of precarious employment. The literature suggests that people in more precarious situations often have smaller, less diverse networks (106); and that, given the tendency to socialize with people of a similar socioeconomic situation (107), networks of people in precarious situations also tend to be less capable of providing resources. In addition, the social capital in support networks is clearly distributed unevenly along the axes of inequality: such as gender, socioeconomic status, age or ethnic/migration background. Previous research has shown that the impact of disruptive socio-labor events such as unemployment has an unequal effect on social networks and their ability to provide social support depending on socioeconomic background, with the networks of people better positioned in the social structure being more resilient (108). Considering the multiple and somewhat contradicting evidence, we see the need to further delve into the capacity of support networks to buffer the impacts of precarious employment on stress and health.

In short, numerous studies have shown that these four aspects arising from precarious employment (i.e., degradation of working conditions, material deprivation, subjective experience of uncertainty, and weakening of informal support networks) are powerful explanatory factors of health and disease (95, 109–111), with potentially important synergistic effects among them. However, no study has analyzed precarious employment jointly with the resulting complex web of socioeconomic factors to explain its link to health, and, more specifically, stress.

The following diagram graphically summarizes the theoretical model described above, whereby the authors hypothesize that precarious employment can have a significant effect on stress, health and the quality of life of working people.

### Objectives

Building upon the research challenges identified, the PRESSED project has three main objectives:

1. To analyze the associations between multidimensional precarious employment and chronic stress among salaried workers in Barcelona, measured both subjectively and using biological indicators.
2. To improve our understanding of the pathways and mechanisms linking precarious employment with stress, health, and well-being.
3. To analyze health inequalities by gender, social class, and place of origin for the first two objectives.

## Methods and Analyses

### General Research Design

**Figure 2:**
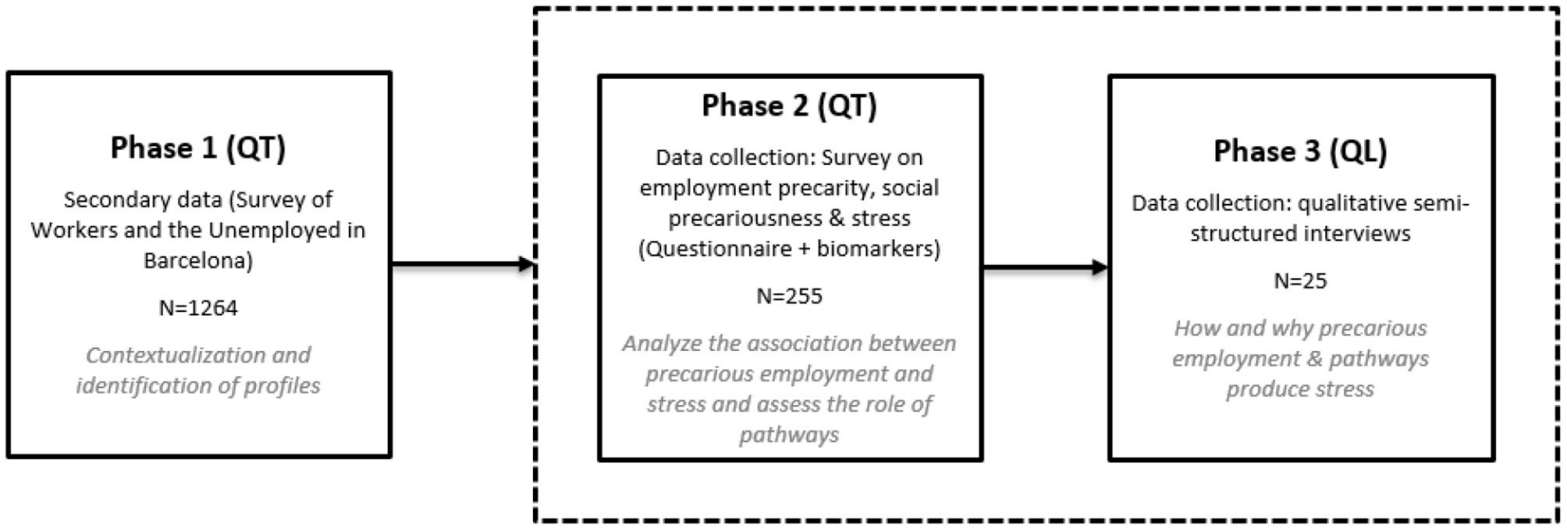
Flowchart of the PRESSED project study design. Sequentiality and interpretive integration of the phases, methods, sample size and objectives. Source: authors' own Phases 1 and 2 were conducted between 2018 and 2020, while Phase 3 will be conducted in 2021.

The proposed research has a three-phase sequential mixed design (112): quantitative-quantitative-qualitative (Figure 2). In this type of methodological design, each phase has a different sample size and collects different types of data. These phases are strung together so that the partial results of the first phases can guide the sample selection of the subsequent phases. Thus, the study population (i.e., salaried workers aged between 24 and 60 years in Barcelona) is studied by means of three different methodological strategies that combine random and purposive sampling logics. This form of “mixed method sequential sampling” (113) acts as the basis for the transferability or generalization of the data obtained from the specific context to the wider context. Therefore, it enables conducting a feasible, realistic project. As noted by Verd and López-Roldan (114), it maximizes both the methodological and theoretical efficiency of the project. As regards the former, the fact that the results of the most extensive data allow selecting cases that are the subject of more intensive analyses amplifies the object of study and grants robustness to the processes of inference, transferability, and generalization of results. As for the latter, integrating the different phases allows providing a comprehensive explanation of how patterns of association between precarious employment and stress are built.

### Phase 1: Current Status and Social Map of Precarious Employment Using Secondary Data

This phase was dedicated to reviewing the existing literature relevant to the research objectives and to developing the “social map of precarious employment” drawing on secondary data, specifically the Survey on Workers and the Unemployed of Barcelona (EPYPB, 2017-2018). The EPYPB (*N* = 1,264) was particularly useful for this phase as it aimed at studying in depth the diversity of precarious employment situations and the resulting social processes in the city of Barcelona. As a reference population it took individuals of legal working age, living in the city of Barcelona and who had worked at least1 h during the reference period. For further details of the survey see (115).

The main objective of this phase of analysis was to identify the prevalence and distribution of multidimensional precarious employment among the wage-earning population and the most affected social profiles. The results contextualized and framed the subsequent phase's results establishing the scope of Phase 2 at a population level, according to the general map drawn in Phase 1. This theoretical and empirical work allowed refining the project's model and methodological design.

### Phase 2: Survey on Precarious Employment, Pathways of Precarization and Stress

This phase involved conducting a survey on precarious employment, social pathways, and stress on a smaller, tailored sample (*N* = 255) in order to examine the relationship between precarious employment and individuals' stress levels as a potential predictor of physical and mental health, and to identify the role of the hypothesized pathways (Figure 1) as explanatory factors of this relationship. In this survey, participants' perception of their stress level was measured subjectively through questionnaires, and objectively through the quantification of cortisol levels in their hair and other compounds related to HPA and the gonadal axis as biological indicators of chronic stress.

#### Pressed Survey Sample

Although the sample of this survey is small and not representative, its interest lies in the internal variability and comparability of results. In other words, it is designed to show how cortisol in hair (and other related biomarkers of the HPA axis) is distributed throughout the whole range of experiences of precarious employment identified in the previous phase, and to unravel the explanatory power of the pathways and mechanisms of interest. On the other hand, this sample size, under the assumptions of an alpha risk of 0.05 and a beta risk below 0.2 (80% power) in a two-sided test and with a sample loss rate of 0%, will allow us to estimate correlation coefficients of 0.175.

Accordingly, a non-probabilistic sampling strategy was implemented, based on proportional quotas by sex, age group (25–34 years vs. 35–60 years), place of origin (born in Spain vs. born abroad) and socioeconomic level of district of residence (medium, medium-high or high vs. medium-low and low-income districts) (see Table 1). Participants were recruited from the pool of participants in the 2017 Barcelona Health Survey within the selected age range who had agreed to being contacted again for future studies and agreed specifically to being contacted by the University for this project (*n* = 1,210). Also, in order to offset the bias of this subsample toward profiles with higher levels of education and income, the abovementioned recruitment strategy was complemented with 40 individuals contacted through social and labor organizations that work with groups of precarious workers and a slight overrepresentation of immigrant women. Interviewees were rewarded for their participation with a courtesy gift of 30€.

**Table 1:** Characteristics of the PRESSED survey sample.

	**Born abroad**		**Born in Spain**		**Total**
	**Age 20–34**	**Age 35–60**	**Age 20–34**	**Age 35–60**	
	**Medium-high or high income districts**				
Women	4	10	11	31	56
Men	2	8	11	28	49
	**Medium-low or low income districts**				
Women	6	13	12	43	74
Men	7	12	14	43	76
Total	19	43	48	145	255

Inclusion criteria were: (i) being a salaried worker or bogus self-employed worker serving a single employer, (ii) being between 24 and 60 years old, (iii) living independently in Barcelona (i.e., young people living with their parents were excluded), (iv) the length of hair at the back of the head being of at least one centimeter, and (v) not having taken holidays within the month prior to the interview. Exclusion criteria were: (i) having taken corticosteroids within the month prior to the interview, (ii) being diagnosed with an adrenal disease, and (iii) being pregnant, due to possible alterations in cortisol levels.

#### Questionnaire, Indicators, and Variables

The questionnaire, requiring ~40 min to complete, included questions on the different topics of interest of the study as well as standard questions on sociodemographic characteristics. Below we set out how the concepts of precarious employment, working conditions, poverty, uncertainty, social support networks, and stress were operationalized and recorded (for further details on the questions included in the questionnaire, see Table 2 and the full questionnaire in Spanish, in the Supplementary Material).

**Table 2:** Detail of the variables included in the PRESSED survey questionnaire.

**Control**	Surveyor ID	Questionnaire number	Interview date and time (start, end)				
**Sociodemo-graphics**	Sex	Date of birth	Place of birth (other country: which?)	Nationality	District of residence	Level of education	
**Occupational (descriptive)**	Company activity	Occupation	Additional jobs	Company size	Supervision	Existence of trade unions	
**Employment precariety**	**Working day**	Contracted and real hours	Preferred working hours	Overtime Volunteering? Compensation?	Working days/month longer than 10h, weekends	Advanced notice of schedule changes	Type of working day
	**EPRES**	**Temporariness** Contract type, Duration, Tenure	**Wages:** Categories scale and no. of annual payments; Covers needs, Unforeseen expenses	**Disempowerment:** Working day decision, Wages decision	**Vulnerability:** For complaining, powerlessness, dismissal, authoritarian treatment, replaceable	**Rights:** Parenthood, pensions, unemployment compensation	**Exercise of rights:** Days off, holidays, family leave, personal leave, sick leave
	**Various 2^nd^ job insecurity**	Supplementary jobs?	Actual hours	Contract type	Seasonality		
**Working Conditions**	**Psychosocial risks**	Contents and requirements (7 items)	Active work, influence and development (6 items)	Social support in the workplace (8 items)	Leadership quality (6 items)	Esteem (2 items)	
	**Other occupational risks**	Discrimination, violence (2 items)					
**Domestic workload**	Double burden (4 items)	Proportion of household chores assumed	Domestic chores outsourced	Care workload + hours of dedication			
**Life conditions**	**Risk of poverty**	Household make-up: no. of people, ages	Months and hours worked prev. year (respondent and household members)	*Household income* (all members, all sources) (open, if no answer: bracket + Threshold)			
	**Material deprivation**	9 AROPE items + leisure, internet, computer	Getting to the end of the month				
**Uncertainty/ Insecurity**	**Job insecurity**	Emotional insecurity (job, labor market, hours, wages)	Cognitive insecurity (job, labor market, hours, wages)				
	**Financial insecurity**	How long could be without wages					
	**Agency**	Ability to cover a major expenditure	Postpone projects or major changes	Ability to make future plans			
	**Trajectory**	Unemployment	Unemployment in last 10 years	Total duration of unemployment 10 years	Length of last event Unemployment	Age 1^st^ employment	
		Instability	No. contracts last 3 years	Indef. contract last 3 years	Informal last 3 years		
**Networks**	**Participation in orgs.**	Type	Intensity	Participation at the workplace			
	**Support**	DUFSSS (emotional support)	Other instrumental support: housing, employment counseling, car, €300	Receive services from NGOs			
	**Employment situation interferes**	In personal and family life	In quality of family life	Prevents doing things with family and friends			
**Stress**	Perceived Stress Scale (PSS)	Stressful life events	Workplace stress (AIS)	Physical symptoms (4 items)	Cognitive symptoms (4 items)	Activities to reduce stress (open)	Biomarkers
**Health**	Self-perceived health	Mental health and well-being (WHO-5)	Height and weight	Medication	Functional limitations	Use and habits: smoking; alcohol; sport	
**Miscellaneous**	Possibility of recontacting	Contact details: phone, mail	Comments	Incidents			

##### Employment Precariousness

EPRES, which consists of 22 items structured into the abovementioned six dimensions: temporariness, wages, rights, capacity to exercise rights, disempowerment, and vulnerability to authoritarian treatment. To further describe the job of the interviewee other questions were included such as occupational status, sector of activity, company size, and the performance of supervisory tasks. Finally, several questions related to the working day (such as doing overtime, involuntary part-time work, the ability to take breaks, etc.) were also added.

##### Working Conditions

Psychosocial risks. A selection of questions was included from the CoPsoQ questionnaire (version 3) (116), which includes items on job contents and requirements; active work, influence and performance; social support in the workplace; quality of leadership; and esteem.

##### Working Poverty

The questionnaire followed the AROPE methodology (117), which relates to the financial situation of the household and takes three forms of poverty into account: work intensity of household members, material deprivation, and relative poverty. To this end, the household composition and the labor participation of all its members during the year prior to the interview were reconstructed. Interviewees were asked about the joint income of all of the household members, taking into account income from work as well as social benefits and other patrimonial revenues to calculate the equivalized disposable income. This indicator is calculated considering household members as consumer units whose weighting varies according to the number of household members and their ages (1 to the first adult, 0.5 to the second and each subsequent person aged 14 and over, and 0.3 to each child aged under 14). In OECD countries, people living in households at risk of relative poverty are commonly defined as having an income of <60% of the median wage. The reference community considered for this study is that of Barcelona, whose poverty line threshold for a unipersonal household, according to the 2016 and 2017 survey on living conditions, was 11,122.40 euros per annum (926.90 euros per month).

To measure material deprivation [considered by Crettaz (118) a more sensitive indicator in the lower part of income distribution], the subjects were asked about the availability of items included in the AROPE battery, alongside three further items related to the availability of internet, computer, and leisure.

##### Uncertainty

This item was collected using information concerning various aspects. First, perceived job insecurity. Perceived insecurity measures include measures oriented toward the perceived likelihood of the occurrence of an event (cognitive insecurity) and the emotional assessment or worry that this event causes (emotional insecurity). Both measures are applied to three domains of job insecurity: losing employment (job loss insecurity), finding alternative employment (labor market insecurity), and the worsening of working conditions (working conditions insecurity) (80). Second, financial insecurity was included, measured through the amount of time the interviewee would be able to live without income before being in a serious financial situation. Third, capacity of exerting agency, which involved three questions inspired by PEPÍN (119) concerning the existence of limitations for major expenditure, personal projects or future plans due to the employment situation. Finally, the previous labor market trajectory was considered; in particular, the experience and duration of both unemployment and unstable or informal employment prior to the interview.

##### Social Support Networks

The Duke-UNC Functional Social Support scale (DUFSS) was used, which measures perceived functional social support, basically emotional support in the affective and confidential subdimensions (120). This scale has also been validated for the case of Spain (121). This information was supplemented with four items of perceived availability of instrumental support, inspired by the Interpersonal Support Evaluation List (ISEL) questionnaire (122), as well as with questions concerning participation in civil society associations or organizations that provide support to citizens.

##### Perceived Stress

The questionnaire measured perceived stress in several ways. In the first place, the questionnaire included the Spanish 2.0 version of the Perceived Stress Scale (PSS), based on the full 14-item version by Cohen (123), and adapted by Sanz-Carrillo et al. (124). In the second place, a question regarding workplace stress (125) was also used. Finally, the questionnaire included items concerning physical and cognitive symptoms of stress included in the CoPsoQ questionnaire, as well as open questions about the existence of stressful events in the previous month and the performance of activities to reduce stress.

##### Health

The questionnaire included questions about mental health and well-being using the WHO-5 scale (126), self-perceived health, the subject's height and weight to calculate body mass index, the existence of functional limitations, and healthy habits relating to tobacco and alcohol consumption and to physical activity.

#### Hair Sampling and Analysis Procedure

Upon completing the questionnaire, a sample of participants' hair equivalent to a lock of hair of the thickness of a pen (between ~30 and 50 mg) was taken from the back of the head using scissors cutting as close to the skin as possible. The samples were taken by the interviewers, who were trained to that end.

The first centimeter of the lock of hair that is in contact with the scalp is the biological material subjected to laboratory analysis. Since hair grows about one centimeter per month (127), the selection of this segment implies that the level of chronic stress accumulated over the month prior to sampling can be identified. The analysis was carried out using a LC-MS/MS (liquid chromatography-tandem mass spectrometry) validated method (128). From this analysis, 11 targeted compounds were quantified (see Table 3). In particular, eight of them belong to the HPA axis, which is considered to play a central role in the physiological response to stress. Cortisol and its metabolites were included, as well as other related compounds.

**Table 3:** List of biomarkers of the hypothalamic-pituitary-adrenal axis (HPA) and gonadal axis obtained by LC-MS/MS analysis of hair samples (abbreviation and full name).

**Abbreviations**	**Biomarker name**
F	Cortisol
20αDHF	20α-dihydrocortisol
20βDHF	20β-dihydrocortisol
E	Cortisone
20αDHE	20α-dihydrocortisone
20βDHE	20β-dihydrocortisone
βCortolone	β-cortolone
A	11-dehydrocorticosterone
T	Testosterone
AED	Androstendione
Prog	Progesterone

#### Data Analysis

It is planned to perform partial analyses using linear regressions to delve into the association between precarious employment, the different pathways and indicators of stress, and well-being. It is also planned to perform mediation analysis with structural equation models to evaluate the project's conceptual model (Figure 1) as a whole. All analyses will be stratified by sex, and biomarker predictors will be controlled, at least, for age and body mass index.

### Phase 3: Qualitative Depth on the Link Between Precarious Employment and Well-Being

Finally, following the logic of an intensive analysis of case studies as a final phase in a mixed-methods sequential investigation, a sub-sample of the previous survey (*N* = 25) will be selected. With this sample, semi-structured qualitative interviews will be conducted to look in greater depth into how and why precarious employment and the associated social precariousness have an impact on stress, health and well-being.

Thus, the aim of this third phase is to deepen qualitatively into the understanding of the particular mechanisms that explain or predict the link between precarious employment and stress considering the complex casuistic of life situations that contextualize each case. This qualitative phase supplements previous results in the sense that it allows, first, identifying the logical relationship established between the dimensions of precarious employment, and stress and health. Following the “process tracing” method of Bennett and Elman (129), work will be carried out with a procedural notion of the observed phenomena. This is a common method used within the qualitative framework of case studies, which have a small sample size and work with a notion of causality not based on correlation and covariance but on identifying mechanisms and processes that connect causes and effects. Second, it also allows obtaining information on difficult aspects to harness via standardized questionnaires. In this sense, the flexible and open nature of semi-structured interviews will allow deepening into the subjective perception of stress and discomfort and the subjective experience of precarious employment and associated pathways of social precariousness. Moreover, qualitative methodologies will be applied in the interview to reconstruct the configuration and changes in social support networks (130). These interviews will be analyzed by means of an interpretive, qualitative content analysis supported by Atlas.ti.

Cases will be recruited from among survey participants who have consented to being re-contacted for the next phase of the study. Criteria for their selection will be based on their level of precarious employment, the type of association shown between precarious employment and stress, and according to the identified role of the explanatory pathways.

Due to the outbreak of the COVID-19 pandemic between survey data collection and the implementation of the qualitative interviews, and the resulting social and economic crises that caused (and are still causing) a major impact on the labor market, the linkage between the qualitative and quantitative phase of the study might be hampered. In order to overcome this issue, we plan to re-contact cases prior to the interview to update or confirm the validity of the data obtained in the survey conducted in the previous phase.

## Discussion

Promoting inter- or transdisciplinary scientific research is often proposed as a way to advance science and to expand its boundaries beyond the objects of study and theoretical and methodological approaches common to each discipline or area of knowledge. However, there are few benchmarks on how to concretize interdisciplinarity in the practical aspects of research projects. Thus, this protocol provides an example of interdisciplinaryty in the approach to and the development of the methodological design of a research project on occupational health. Furthermore, both the interdisciplinary approach, the design of a mixed methodology (combining quantitative and qualitative data) and, especially, the use of biomarkers, are innovative elements in the treatment of this subject, hence the results are expected to be able to provide innovative evidence of relevance to the scientific community.

Moreover, on a more general level this project should contribute to the “paradigm shift” led by the World Health Organization's Commission on Social Determinants of Health in its influential report of 2008, which aims to recognize the real weight of socioeconomic determinants as fundamental causes of health inequalities. Thus, the PRESSED project can help change the view held by society and much of the scientific community on the causes of illness, currently dominated by explanations related to behavioral and biological factors.

Second, this project is expected to have a social and economic impact. In line with the principles established by the 2030 Agenda for Sustainable Development, the project aims to promote sustained, inclusive and sustainable economic growth, full, and productive employment and decent work for all, and therefore help to reduce social inequality and enhance social cohesion. Given that the deterioration of the quality of employment conditions and subsequent precarious employment is a phenomenon gaining importance in the context of post-industrial economies, but especially in the Spanish context of (non)recovery from the Great Recession of 2008 compounded by the impact of the Covid-19 pandemic, there is a need for research projects that provide evidence of the most harmful and unsustainable aspects of this model of employment, and of the pathways and mechanisms whereby it plausibly causes such a negative, inegalitarian impact.

More specifically, the PRESSED project research results are expected to culminate in the proposal of scientifically informed public policies. The project will end with the drafting of a document of recommendations for the design of social, labor, and health policies aimed at refining epidemiological surveillance systems and ultimately improving the health, well-being and quality of life -especially of the most vulnerable workers, and fight the pressing health inequalities that currently exist.

## Ethics and Dissemination

### Ethics

This study was reviewed and approved by the Institutional Commission for Ethics Review of Projects (CIREP) of Universitat Pompeu Fabra (UPF) under CIREP nr.0079. Written informed consent was obtained from all participants prior to participating in the study, and participants were reminded that they can withdraw at any time. All data used in this project are duly processed, preserving data anonymity and confidentiality, fulfilling all legal and ethical requirements, and in compliance with the laws of Spain governing personal data protection.

### Dissemination

The PRESSED project has an engagement, dissemination, and impact plan in order to maximize the social outreach and transfer of results. Its dissemination strategy aims to reach academia, stakeholders, policy makers and the general public. First, academia, by publishing scientific articles in specialized peer-reviewed journals, conference papers and reports on the website of the GREDS-EMCONET research group and in the UPF e-Repository. Second, stakeholders, policy makers and the general public through informative reports setting out the results, and press releases in the media and social networks. By the end of the project, a conference will be organized to present and discuss the findings, which will be open to and promoted among stakeholders.

In order to strengthen social outreach and the transfer of results to the second target audience, the PRESSED project has aroused the interest of three institutions that have formally expressed their interest in the results of the project. The following are PRESSED project Observer Promoter Entities (EPOs): Institut de Seguretat i Salut Laboral (ICSSL - Institute of Occupational Safety and Health) of the Generalitat de Catalunya (Government of Catalonia), European Trade Union Institute (ETUI), and Department of Public Health, Environmental, and Social Determinants of Health of the World Health Organization (WHO).

Other social organizations and public health entities are expected to collaborate both in attracting participants for the study and in disseminating the research results: Public Health Agency of Barcelona (ASPB), trade unions and social associations, notably the trade union Comisiones Obreras de Cataluña (CCOO - Workers' Commissions), the Confederació General de Treball a Catalonia (CGT - General Confederation of Labor), and social movements such as No+Precariedad (“No more precarity”), Las Kellys (“The Maids”), and Mujeres Migrantes Diversas (“Diverse Migrant Women”), among many others.

## Ethics Statement

The studies involving human participants were reviewed and approved by Institutional Commission for Ethics Review of Projects (CIREP) of Universitat Pompeu Fabra (UPF). The patients/participants provided their written informed consent to participate in this study.

## Author Contributions

MB and MJ are PIs of the project and have led the writing of the manuscript. FB, PJ, FM, XB-R, OP, AG-G, AV, EP, and JB have provided theoretical and technical advice and have revised and accepted the manuscript. All authors contributed to the article and approved the submitted version.

## Conflict of Interest

The authors declare that the research was conducted in the absence of any commercial or financial relationships that could be construed as a potential conflict of interest.
