# Supplementary material for: Precarious Employment and Stress: The Biomedical Embodiment of Social Factors. PRESSED Project Study Protocol
Source: Front Public Health. 2021 Mar 30;9:649447. doi: 10.3389/fpubh.2021.649447 (PMC8042135; doi:10.3389/fpubh.2021.649447)
Supplement: Supplementary file 1 [file Data_Sheet_1.PDF]

PRESSED - Precariedad laboral y estrés

Control

Page description:

ID 3

1. Código entrevistador/a *

- 01- Fabricio Mendez
- 02- Maria Granja
- 03- Eva Padró
- 04- Blanca Paniello
- 05- César-Darío Chapparo
- 06- Júlia Armengol
- 07- Mireia Julià
- 08- Mireia Bolíbar
- 09- David Vílchez
- 10- Irene Galí
- 11- Eva Padrosa
- 12- Neus Artés
- 13- Laia Ollé

ID 4

2. Número de encuesta *

ID 6

3. Fecha de la entrevista (DD/MM/AAAA) *

ID 7

4. Hora de la entrevista (HH:MM formato 24h) *

ID 5

5. Distrito donde vive el entrevistado *

- Sarrià- Sant Gervasi
- Horta- Guinardó
- Nou Barris
- Sant Andreu
- Gràcia
- Les Corts
- Eixample
- Sant Martí
- Ciutat Vella
- Sants- Montjuïc

Sociodemográficas

Page description:
Para empezar le haré algunas preguntas sociodemogràficas

ID 14

6. Sexo (no leer) *

- Hombre
- Mujer
- Otros

ID 15

7. ¿Cuál es su fecha de nacimiento completa, día, mes y año? (DD/MM/AAAA) Tiene que haber nacido mínimo en el 1995 y máximo en el 1959 *

ID 16

8. Nacionalidad
*

- Española
- Española y otra extranjera
- Extranjera
- NS (no leer)
- NC (no leer)

ID 17

9. ¿Dónde nació usted? *

- Barcelona
- Otro municipio de Cataluña
- Otra Comunidad Autónoma
- Otro país
- NS (no leer)
- NC (no leer)

18

10. ¿Cuál es su nivel máximo de estudios finalizados?
MOSTRAR TARJETA **1** *

- 1. No sabe leer ni escribir
- 2. Estudios primarios incompletos (sabe leer y escribir sin haber finalizado la educación primaria)
- 3. Estudios primarios completos: primaria completa (LOGSE) o cinco cursos aprobados de EGB o programas de garantía social o PQPI (programa de cualificación profesional inicial o ESO incompleta
- 4. Primera etapa de educación secundaria: graduado escolar, bachillerato elemental, EGB o ESO completa, haber aprobado las pruebas de acceso a ciclos formativos de grado medio
- 5. Enseñanza de bachillerato: bachillerato superior, BUP, bachillerato del plan nuevo (LOGSE), PREU o COU o prueba de acceso a la universidad para mayores de 25 años
- 6. Formación profesional de grado medio: oficial industrial, FP I, ciclos formativos de grado medio
- 7. Formación profesional de grado superior: maestría industrial, FP II, ciclos formativos de grado superior, estudios de artes aplicadas y oficios
- 8. Estudios universitarios de primer ciclo: diplomaturas universitaria, arquitectura e ingeniería técnica, enseñanza universitaria de ciclo corto y enseñanza universitaria de primer ciclo (3 cursos)
- 9. Estudios universitarios de segundo grado:(licenciatura, arquitectura, ingeniería y grados
- 10. Estudios universitarios de doctorado, posgrado, master, MIR o equivalente
- 11. Otra posibilidad . Específíquela
- NS (no leer)
- NC (no leer)

Ocupacionales (descriptivo)

Page description:
Las siguientes preguntas estan relacionadas con su trabajo principal

ID 25

11. ¿Cuál es la actividad principal de la empresa donde trabaja? (respuesta abierta) *

ID 26

12. ¿Qué trabajo en concreto realiza en esta empresa? (Respuesta abierta) *

ID 27

13. Supervisa usted directamente a alguien en su trabajo? En caso afirmativo, de cuantas personas? *

- No
- Sí, de 1 a 4 personas
- Sí, de 5 a 10 personas
- Sí, de 11 a 20 personas
- Sí, más de 20 persones
- NS (no leer)
- NC (no leer)

ID 28

14. ¿Cuántas personas trabajan en su centro de trabajo? (Sólo en dicho centro) *

- 1 (solo el entrevistado)
- 2-5
- 6-9
- 10-49
- 50-99
- 100-249
- 250 o más
- NS (no leer)
- NC (no leer)

Precariedad

Page description:

VALIDATION Must be numeric

ID 33

15. ¿De cuantas horas a la semana es el contrato en su principal trabajo remunerado? *

ID 34

16. Habitualmente, ¿cuántas horas a la semana trabaja usted realmente en su principal trabajo remunerado? *

- Horas reales
- NS (no leer)
- NC (no leer)

LOGIC Show/hide trigger exists.

ID 336

17. (Para el encuestador/a: coinciden las respuestas de las horas trabajadas y las del contrato?)
*

- Sí
- No

ID 35

18. Suponiendo que pudiera elegir libremente sobre sus horas de trabajo y teniendo en cuenta la necesidad de ganarse la vida: ¿cuántas horas semanales preferiría trabajar actualmente? *

Horas

Minutos

LOGIC Hidden unless: #17 Question "(Para el encuestador/a: coinciden las respuestas de las horas trabajadas y las del contrato?)
" is one of the following answers ("No")

ID 37

19. Las horas extra, las ha realizado voluntariamente o involuntariamente?

- Voluntariamente
- Involuntariamente (obligado por la empresa)
- NS (no leer)
- NC (no leer)

LOGIC Hidden unless: #17 Question "(Para el encuestador/a: coinciden las respuestas de las horas trabajadas y las del contrato?)
" is one of the following answers ("No")

ID 38

20. Dichas horas extra han sido compensadas o no?

- No compensadas
- Compensadas monetariamente
- Compensadas por horas ordinarias y descansos
- Otras formas de compensación
- NS (no leer)
- NC (no leer)

VALIDATION Must be numeric

ID 40

21. Habitualmente, ¿cuántas veces al mes hace jornadas de más de 10 horas? (si no hace ninguna, poner 0)
*

ID 42

22. ¿Se producen con frecuencia cambios en su horario de trabajo? En caso afirmativo, ¿con cuánto tiempo de antelación se le informa de dichos cambios? *

- No
- Sí, con unas semanas de antelación
- Sí, unos días antes
- Sí, el día antes
- Sí, el mismo día
- NS (no leer)
- NC (no leer)

VALIDATION Must be numeric

ID 44

23. ¿Cuántos días al mes trabaja en fines de semana y/o festivos? (número de 0 a 8) *

ID 46

24. ¿Qué tipo de jornada u horario (ordinario, establecido) tiene en su trabajo? MOSTRAR TARJETA **3** *

- Jornada partida (mañana y tarde)
- Jornada continua, por la mañana (ej: de 8 a 15 horas)
- Jornada continua, por la tarde (ex. de 13 a 21 horas)
- Jornada continua, por la noche (entre las 22 y las 6 horas)
- Turnos rotativos, excepto el de noche
- Turnos rotativos, incluyendo el de la noche
- Jornada irregular o variable según los días
- Disponibilidad según las necesidades de la empresa
- Otra posibilidad. Especifíquela:
- NS (no leer)
- NC (no leer)

LOGIC Show/hide trigger exists.

ID 48

25. ¿Qué tipo de relación laboral tiene ahora en esta empresa?
(Nota: En las administraciones públicas trabajan funcionarios y laborales; los laborales han de ubicarse en las opciones de contrato temporal o indefinido) MOSTRAR TARJETA **2** *

- Tengo un contrato indefinido de fijo discontinuo
- Tengo un contrato indefinido
- Soy funcionario de carrera
- Soy funcionario interino
- Tengo un contrato temporal formativo (de aprendizaje, formación, en prácticas, becario/a ....)
- Tengo un contrato temporal (contrato por obra y servicio, circunstancias de la producción, de interinidad ...)
- Aún siendo autónomo, en realidad trabajo para alguien del que dependo, trabajo para uno o dos clientes (soy un trade: trabajador autónomo dependiente)
- Trabajo sin ningún tipo de contrato
- NS (no leer)
- NC (no leer)

LOGIC Show/hide trigger exists. Hidden unless: #25 Question "¿Qué tipo de relación laboral tiene ahora en esta empresa?
(Nota: En las administraciones públicas trabajan funcionarios y laborales; los laborales han de ubicarse en las opciones de contrato temporal o indefinido)
MOSTRAR TARJETA **2**" is one of the following answers ("Soy funcionario interino","Tengo un contrato temporal formativo (de aprendizaje, formación, en prácticas, becario/a ....)","Tengo un contrato temporal (contrato por obra y servicio, circunstancias de la producción, de interinidad ...)","Aún siendo autónomo, en realidad trabajo para alguien del que dependo, trabajo para uno o dos clientes (soy un trade: trabajador autónomo dependiente)","Trabajo sin ningún tipo de contrato","NS (no leer)","NC (no leer)")

ID 49

26. ¿Cuál de estas opciones se ajusta más a su situación personal?

- Tengo un contrato temporal que NO PRECISA fecha de finalización
- Tengo un contrato temporal que PRECISA fecha de finalización
- NS (no leer)
- NC (no leer)

LOGIC Hidden unless: #26 Question "¿Cuál de estas opciones se ajusta más a su situación personal?" is one of the following answers ("Tengo un contrato temporal que PRECISA fecha de finalización")

ID 50

27. ¿Cuál es la duración de su contrato temporal actual? (ANOTAR 0 EN LA CELDA QUE NO PROCEDA)

Tengo un contrato temporal de.... *

- [ ] Nº de DIAS (si procede) : ESCRIBA LA RESPUESTA EN EL RANGO 1 – 31
- [ ] Nº de MESES (si procede): ESCRIBA LA RESPUESTA EN EL RANGO 1 – 11
- [ ] Nº de AÑOS (si procede): ESCRIBA LA RESPUESTA EN EL RANGO 1
- [ ] NS (no leer)
- [ ] NC (no leer)

ID 51

28. ¿Cuánto tiempo en total lleva trabajando para esta empresa? (ANOTAR 0 EN LA CELDA QUE NO PROCEDA) *

	Nº de DIAS (si procede) :ESCRIBA LA RESPUESTA EN EL RANGO 1 – 31
	Nº de MESES (si procede): ESCRIBA LA RESPUESTA EN EL RANGO 1 – 11
	Nº de AÑOS (si procede): ESCRIBA LA RESPUESTA EN EL RANGO 1
	NS (no leer)
	NS (no leer)

ID 53

29. Aproximadamente, ¿cuánto cobra usted neto al mes? MOSTRAR TARJETA **4**

*

- 1) 300 euros o menos
- 2) Entre 301 y 450 euros
- 3) Entre 451 y 600 euros
- 4) Entre 601 y 750 euros
- 5) Entre 751 y 999 euros
- 6) Entre 1.000 y 1.200 euros
- 7) Entre 1.201 y 1.500 euros
- 8) Entre 1.501 y 1.800 euros
- 9) Entre 1.801 y 2.100 euros
- 10) Entre 2.101 y 2.400 euros
- 11) Entre 2.401 y 2.700 euros
- 12) Entre 2.701 y 3.000 euros
- 13) Más de 3.000 euros
- NS (no leer)
- NC (no leer)

ID 337

30. ¿Cuantas pagas recibe al año? (12 en el caso que tengan las pagas extra prorrateadas. Lo más habitual es tener 14 pagas)

*

- .
- NS (No leer)
- NC (No leer)

ID 57

31. ¿Con qué frecuencia su salario actual ....?
MOSTRAR TARJETA **5**
*

	Siempre	Muchas veces	Algunas veces	Solo alguna vez	Nunca	NS (no leer)	NC (no leer)
Le permite cubrir sus necesidades básicas cotidianas	○	○	○	○	○	○	○
Le permite cubrir los gastos imprevistos de importancia	○	○	○	○	○	○	○

ID 314

32. Cuanto tiempo podría estar sin su salario? *

- ○ Menos de 2 meses
- ○ Entre 2 meses y 1 año
- ○ Más de 1 año
- ○ NS (no leer)
- ○ NC (no leer)

63

33. ¿Cómo se decidieron las siguientes condiciones de trabajo en relación a su jornada?
Leer opciones
*

- Se ajusta estrictamente a lo establecido en el convenio colectivo (de sector, de empresa...) o pacto de empresa o acuerdo (en el caso de la función pública)
- De forma unilateral la dirección de la empresa o mis superiores deciden mejoras sobre el convenio
- De forma unilateral lo decide la dirección de la empresa o mis superiores, al margen del convenio
- A partir del acuerdo entre mis superiores y yo mismo
- En mi equipo de trabajo
- NS (No leer)
- NC (No leer)

64

34. ¿Y en relación a su salario? Leer opciones *

- Se ajusta estrictamente a lo establecido en el convenio colectivo (de sector, de empresa...) o pacto de empresa o acuerdo (en el caso de la función pública)
- De forma unilateral la dirección de la empresa o mis superiores deciden mejoras sobre el convenio
- De forma unilateral lo decide la dirección de la empresa o mis superiores, al margen del convenio
- A partir del acuerdo entre mis superiores y yo mismo
- En mi equipo de trabajo
- NS (No leer)
- NC (No leer)

66

35. Indique ¿con qué frecuencia en esta empresa .....

MOSTRAR TARJETA **5** *

	Siempre	Muchas veces	Algunas veces	Solo alguna vez	Nunca	NS (no leer)	NC (no leer)
Tiene miedo de reclamar mejores condiciones de trabajo	○	○	○	○	○	○	○
Está indefenso ante el trato injusto de sus superiores	○	○	○	○	○	○	○
Le despedirían de su trabajo si no hiciera todo lo que le piden	○	○	○	○	○	○	○
Le tratan de forma autoritaria	○	○	○	○	○	○	○
Le hacen sentir que usted puede ser fácilmente reemplazado/a	○	○	○	○	○	○	○

73

36. De las siguientes prestaciones que le menciono a continuación, dígame para cada una de ellas si tiene usted derecho a ella o no.

	Sí	No	NS (No leer)	NC (No leer)
Maternidad/paternidad	○	○	○	○
Pensión por jubilación, incapacidad	○	○	○	○
Desempleo (subsidio de desempleo)	○	○	○	○

ID 82

37. ¿Tiene usted derecho a indemnización por despido? *

- Si
- No
- NS (No leer)
- NC (No leer)

ID 83

38. Dígame, ¿con qué frecuencia en esta empresa puede ejercer los siguientes derechos?
MOSTRAR TARJETA **5** *

	Siempre	Muchas veces	Algunas veces	Solo alguna vez	Nunca	NS (no leer)	NC (no leer)
Hacer los días de fiesta semanales sin problemas							
Hacer los días de vacaciones sin problemas							
Coger un día de permiso por motivos familiares (cuidado de menores, personas dependientes, enfermas ...) sin problemas							
Coger un día de permiso por motivos personales (estudios ....) sin problemas							
Coger la baja por enfermedad sin problemas							
Ir al médico cuando lo necesita							

Condiciones de trabajo

Page description:
A. CONTENIDOS Y EXIGENCIAS

ID 91

39. A continuación le haré unas preguntas sobre las exigencias de su trabajo actual. Dígame con qué frecuencia....
MOSTRAR TARJETA **5** *

	Siempre	Muchas veces	Algunas veces	Solo alguna vez	Nunca	NS (no leer)	NC (no leer)
Le resulta imposible terminar sus tareas?	○	○	○	○	○	○	○
Tiene suficiente tiempo para terminar su trabajo?	○	○	○	○	○	○	○
Tiene que trabajar muy deprisa?	○	○	○	○	○	○	○
Tiene que ocuparse de los problemas personales de otras personas?	○	○	○	○	○	○	○

ID 392

40. Continuando con las exigencias y contenidos de su trabajo, en que medida...?
MOSTRAR TARJETA **6** *

	En gran medida	En buena medida	En cierta medida	En alguna medida	En ninguna medida	NS (no leer)	NC (no leer)
El ritmo de trabajo es alto durante toda la jornada?	○	○	○	○	○	○	○
Su trabajo, en general, es desgastador emocionalmente?	○	○	○	○	○	○	○
Su trabajo requiere que esconda sus emociones?	○	○	○	○	○	○	○

ID 99

41. Estas preguntas tratan sobre el margen de autonomía y los contenidos que tiene su trabajo actual. Dígame con qué frecuencia...

MOSTRAR TARJETA **5** *

	Siempre	Muchas veces	Algunas veces	Solo alguna vez	Nunca	NS (no leer)	NC (no leer)
Tiene mucha influencia sobre las decisiones que afectan a su trabajo?	○	○	○	○	○	○	○
Tiene influencia sobre COMO realiza su trabajo?	○	○	○	○	○	○	○

ID 398

42. Continuando con las posibilidades de desarrollo. Dígame con que medida....?

MOSTRAR TARJETA **6** *

	En gran medida	En buena medida	En cierta medida	En alguna medida	En ninguna medida	NS (no leer)	NC (no leer)
Su trabajo permite que aprenda cosas nuevas?	○	○	○	○	○	○	○
La realización de su trabajo permite que aplique sus habilidades y sus conocimientos?	○	○	○	○	○	○	○
Tiene sentido sus tareas?	○	○	○	○	○	○	○
Las tareas que hace, le parecen importantes?	○	○	○	○	○	○	○

403

43. Estas preguntas tratan del grado de definición de sus tareas. En que medida...?

MOSTRAR TARJETA **6** *

	En gran medida	En buena medida	En cierta medida	En alguna medida	En ninguna medida	NS (no leer)	NC (no leer)
Su trabajo tiene los objetivos claros?	○	○	○	○	○	○	○
Sabe exactamente qué se espera de usted en el trabajo?	○	○	○	○	○	○	○
Se le exigen cosas contradictorias en el trabajo?	○	○	○	○	○	○	○
Tiene que hacer tareas que tendrían que hacerse de otra forma?	○	○	○	○	○	○	○
En su empresa, se le informa con suficiente antelación de decisiones importantes, cambios o proyectos de futuro?	○	○	○	○	○	○	○
Recibe toda la información que necesita para hacer bien su trabajo?	○	○	○	○	○	○	○

410

44. En que medida puede afirmar que su superior inmediato...?

MOSTRAR TARJETA **6** *

	En gran medida	En buena medida	En cierta medida	En alguna medida	En ninguna medida	NC (no leer)	NS (no leer)
... planifica bien su trabajo?	○	○	○	○	○	○	○
... resuelve bien los conflictos?	○	○	○	○	○	○	○

415

45. Las siguientes preguntas hacen referencia al reconocimiento en el trabajo. En que medida....?

MOSTRAR TARJETA **6** *

	En gran medida	En buena medida	En cierta medida	En alguna medida	En ninguna medida	NS (no leer)	NC (no leer)
Su trabajo es valorado por la dirección?	○	○	○	○	○	○	○
En su trabajo, recibe un trato justo?	○	○	○	○	○	○	○

419

46. En relación a su trabajo en general, cual es el grado de satisfacción en relación a...?

MOSTRAR TARJETA **7** *

	Muy satisfecho	Bastante satisfecho	Más o menos	Poco satisfecho	Nada satisfecho	NS (no leer)	NC (no leer)
.... sus perspectivas laborales?	○	○	○	○	○	○	○
.... su trabajo teniendo todo en consideración?	○	○	○	○	○	○	○
.... su salario?	○	○	○	○	○	○	○

110

47. Estas preguntas tratan sobre los conflictos y de la relación con sus compañeros y sus jefes inmediatos en el trabajo. Dígame, con que frecuencia...?

MOSTRAR TARJETA **5** *

	Siempre	Muchas veces	Algunas veces	Solo alguna vez	Nunca	NS (no leer)	NC (no leer)
Recibe ayuda y apoyo de su superior inmediato, en caso de necesitarlo?	○	○	○	○	○	○	○
Su superior inmediato habla con usted sobre como hace su trabajo?	○	○	○	○	○	○	○
Recibe ayuda o apoyo de sus compañeros de trabajo, en caso de necesitarlo?	○	○	○	○	○	○	○
Sus compañeros hablan con usted sobre como hace su trabajo?	○	○	○	○	○	○	○
Tiene un buen ambiente con sus compañeros de trabajo?	○	○	○	○	○	○	○
En el trabajo, siente que forma parte de un grupo?	○	○	○	○	○	○	○

127

48. Las preguntas a continuación se refieren a si su trabajo afecta a la vida personal y familiar. Dígame en que medida....?

MOSTRAR TARJETA **6** *

	En gran medida	En buena medida	En cierta medida	En alguna medida	En ninguna media	NS (no leer)	NC (no leer)
Siente que su trabajo consume tanta ENERGIA que perjudica su vida personal y familiar?	○	○	○	○	○	○	○
Siente que su trabajo ocupa tanto TIEMPO que perjudica su vida personal y familiar?	○	○	○	○	○	○	○

423

49. Continuando con como el trabajo afecta en su vida personal y familiar. Dígame con qué frecuencia....?

MOSTRAR TARJETA **5** *

	Siempre	Muchas veces	A veces	Solo alguna vez	Nunca	NS (no leer)	NC (no leer)
Hay momentos en los que necesitaría estar en la empresa y en casa a la vez?	○	○	○	○	○	○	○
Si falta algún día de casa, las tareas domésticas que realiza se quedan sin hacer?	○	○	○	○	○	○	○

126

50. Qué parte del trabajo familiar y doméstico hace usted en relación con los miembros del hogar? *

- ○ Soy el/la principal responsable y hago la mayor parte de las tareas familiares y domésticas
- ○ Hago aproximadamente la mitad de las tareas familiares y domésticas
- ○ Hago más o menos una cuarta parte de las tareas familiares y domésticas
- ○ Solo hago tareas puntuales
- ○ No hago ninguna tarea o casi ninguna
- ○ NS (no leer)
- ○ NC (no leer)

ID 131

51. Tienen en el hogar alguna persona que remunere para realizar taras domésticas como limpiar, planchar, cocinar, etc...? *

- Si
- No
- NS (no leer)
- NC (no leer)

ID 147

52. Indique en que medida observa en su entorno de trabajo (jefes, compañeros, clientes, etc) discriminación por sexo, edad, orígen, discapacidad? *

Nunca	Alguna vez	Algunas veces	Muchas veces	Siempre	NS (no leer)	NC (no leer)
○	○	○	○	○	○	○

ID 467

53. Indique en que medida observa en su entorno de trabajo (jefes, compañeros, clientes, etc) acoso sexual, amenazas o humiliaciones? *

Nunca	Alguna vez	Algunas veces	Muchas veces	Siempre	NS (no leer)	NC (no leer)
○	○	○	○	○	○	○

LOGIC Show/hide trigger exists.

ID 339

54. Además de su trabajo principal remunerado, ¿tiene usted alguna otra ocupación remunerada? *

- Si
- No
- NS (no leer)
- NC (no leer)

LOGIC Hidden unless: #54 Question "Además de su trabajo principal remunerado, ¿tiene usted alguna otra ocupación remunerada?" is one of the following answers ("Si")

ID 340

55. ¿Cuántas horas a la semana de media dedica usted al otro trabajo diferente de su principal trabajo remunerado?

- Número de horas
- NS (no leer)
- NC (no leer)

LOGIC Hidden unless: #54 Question "Además de su trabajo principal remunerado, ¿tiene usted alguna otra ocupación remunerada?" is one of the following answers ("Si")

ID 341

56. ¿Qué tipo de relación laboral tiene ahora con la empresa en este otro trabajo diferente de su principal trabajo remunerado?

ENTREVISTADOR: en las administraciones públicas trabajan funcionarios y laborales; los laborales se tienen que ubicar en las opciones de trabajo indefinido o temporal. Seleccione solo una respuesta.

- Tengo un contrato indefinido de fijo discontinuo
- Tengo un contrato indefinido
- Tengo un contrato temporal formativo (aprendizaje, formación, en prácticas, becario)
- Tengo un contrato temporal (por obra y servicio, circunstancias de la producción, de interinidad)
- Aún siendo autónomo, en realidad trabajo para alguien de quien dependo, trabajo para uno o dos clientes (soy un trade: trabajador autónomo dependiente)
- Profesional o trabajador autónomo
- Trabajo sin ningun tipo de contrato
- Otra situación. Especificar:
- NS (no leer)
- NC (no leer)

LOGIC Hidden unless: #54 Question "Además de su trabajo principal remunerado, ¿tiene usted alguna otra ocupación remunerada?" is one of the following answers ("Si")

ID 342

57. Como es su segunda ocupación?

- Fija
- Temporal
- Estacional
- NS (no leer)
- NC (no leer)

Incertidumbre

Page description:

ID 158

58. Dígame como de probable es que...
MOSTRAR TARJETA **8** *

	Muy probable	Bastante probable	Relativamente probable	Poco probable	Muy poco o nada probable	NS (no leer)	NC (no leer)
Le despidan o no le renueven el contrato	○	○	○	○	○	○	○
Encuentre otro trabajo en caso que se quedara en el paro	○	○	○	○	○	○	○
Le cambien el horario (turno, días de la semana, horas de entrada y salida) contra su voluntad	○	○	○	○	○	○	○
Le bajen el sueldo	○	○	○	○	○	○	○

164

59. Dígame en estos momentos, en qué medida está preocupado/a por...

MOSTRAR TARJETA **6** *

	En gran medida	En buena medida	En cierta medida	En alguna medida	En ninguna medida	NS (no leer)	NC (no leer)
Por si lo despiden o no le renuevan el contrato	○	○	○	○	○	○	○
Por lo difícil que sería encontrar otro trabajo en el caso de que se quedara en el paro	○	○	○	○	○	○	○
Por si le cambian el horario (turno, días de la semana, horas de entrada y salida) en contra de su voluntad	○	○	○	○	○	○	○
Por si le bajan el salario	○	○	○	○	○	○	○

171

60. Considera que su situación laboral.....

MOSTRAR TARJETA **9** *

	Nunca	Alguna vez	A veces	A menudo	Siempre	NS (no leer)	NC (No leer)
Interfiere con su vida personal y familiar?	○	○	○	○	○	○	○
Afecta negativamente a la calidad de vida de su familia?	○	○	○	○	○	○	○
Le impide hacer cosas con la familia y los amigos?	○	○	○	○	○	○	○

382
61. La preocupación por su situación laboral actual ha afectado negativamente la realización de gastos importantes (como compras importantes, cambio de vivienda, educación o actividades de hijos, viajes, reformas, etc.)?
MOSTRAR TARJETA **9** *

- Nunca
- Alguna vez
- A veces
- A menudo
- Siempre
- NS (no leer)
- NC (no leer)

383
62. Dada su situación laboral actual, ha tenido que posponer proyectos personales o cambio familiares o de pareja importantes (como empezar una relación, formalizar una separación, tener hijos o otros cambios familiares)?
MOSTRAR TARJETA **9** *

- Nunca
- Alguna vez
- A veces
- A menudo
- Siempre
- NS (no leer)
- NC (no leer)

384

63. Hasta que punto su situación laboral actual le dificulta hacer planes de futuro a medio plazo, respecto a proyectos personales o familiares?
MOSTRAR TARJETA **9** *

- Nunca
- Alguna vez
- A veces
- A menudo
- Siempre
- NS (no leer)
- NC (no leer)

Estrés

Page description:

199

64. ¿Durante el último año, ha sentido con frecuencia que no puede con su trabajo habitual? *

- Si
- No
- NS (no leer)
- NC (no leer)

181

65. Las preguntas en esta escala hacen referencia a sus sentimientos y pensamientos durante **el último mes**. En cada caso, por favor indique cómo usted se ha sentido o ha pensado en cada situación.
MOSTRAR TARJETA **10** *

	Nunca	Casi nunca	A veces	A menudo	Muy a menudo	NS (no leer)	NC (no leer)

¿con qué frecuencia ha estado afectado por algo que ha ocurrido inesperadamente?	○	○	○	○	○	○	○
¿con qué frecuencia se ha sentido incapaz de controlar las cosas importantes en su vida?	○	○	○	○	○	○	○
¿con qué frecuencia se ha sentido nervioso o estresado?	○	○	○	○	○	○	○
¿con qué frecuencia ha manejado con éxito los pequeños problemas irritantes de la vida?	○	○	○	○	○	○	○
¿con qué frecuencia ha sentido que ha afrontado efectivamente los cambios importantes que han estado ocurriendo en su vida?	○	○	○	○	○	○	○
¿con qué frecuencia ha estado seguro sobre su capacidad para manejar sus problemas personales?	○	○	○	○	○	○	○
¿con qué frecuencia ha sentido que las cosas le van bien?	○	○	○	○	○	○	○
¿con qué frecuencia ha sentido que no podía afrontar todas las cosas que tenía que hacer?	○	○	○	○	○	○	○
¿con qué frecuencia ha podido controlar las dificultades de su vida?	○	○	○	○	○	○	○
¿con que frecuencia se ha sentido que tenia todo bajo control?	○	○	○	○	○	○	○
¿con qué frecuencia ha estado enfadado porque las cosas que le han ocurrido estaban fuera de su control?	○	○	○	○	○	○	○
¿con qué frecuencia ha pensado sobre las cosas que le quedan por hacer?	○	○	○	○	○	○	○
¿con qué frecuencia ha podido controlar la forma de pasar el tiempo?	○	○	○	○	○	○	○
¿con qué frecuencia ha sentido que las dificultades se acumulan tanto que no puede superarlas?	○	○	○	○	○	○	○

196

66. Se ha producido algún hecho importante en su vida en el último mes que le haya preocupado o estresado (como exámenes, mudanzas, problemas familiares, etc)? *

- Si. Podría indicar cuáles?
- No
- NS (no leer)
- NC (no leer)

Salud y calidad de vida

Page description:

201

67. ¿Cómo diría usted que es su salud en general? *

- Muy buena
- Buena
- Regular
- Mala
- Muy mala
- NS (no leer)
- NC (no leer)

VALIDATION Must be numeric

ID 202

68. Aproximadamente, cuantos quilos pesa sin ropa? *

- Quilos
- NS (no leer)
- NC (no leer)

VALIDATION Must be numeric

ID 356

69. Aproximadamente, cuantos centímetros mide sin zapatos? *

- Centímetros
- NS (no leer)
- NC (no leer)

ID 204

70. En los últimos 12 meses, ha tenido algún problema de salud importante que le limitara las actividades durante un mínimo de 15 días? Trabajar, hacer las tareas del hogar o otros *

- Sí
- No
- NS (no leer)
- NC (no leer)

426

71. Las siguientes preguntas hacen referencia a como se ha encontrado durante las últimas 5 semanas. Con qué frecuencia...?

MOSTRAR TARJETA **11** *

	Todo el tiempo	Gran parte del tiempo	Parte del tiempo	Una pequeña parte del tiempo	Nunca	NS (no leer)	NC (no leer)
Ha tenido dolor de estómago?	○	○	○	○	○	○	○
Ha tenido dolor de cabeza?	○	○	○	○	○	○	○
Ha tenido palpitaciones?	○	○	○	○	○	○	○
Ha tenido tensión muscular?	○	○	○	○	○	○	○
Problemas para concentrarse?	○	○	○	○	○	○	○
Problemas para pensar de forma clara?	○	○	○	○	○	○	○
Problemas para tomar decisiones?	○	○	○	○	○	○	○
Problemas para recordarse de las cosas?	○	○	○	○	○	○	○

205

72. Por favor, indique para cada una de las cinco afirmaciones cual define mejor cómo se ha sentido usted durante la últimas dos semanas
MOSTRAR TARJETA **12** *

	Todo el tiempo	La mayor parte del tiempo	Más de la mitad del tiempo	Menos de la mitad del tiempo	De vez en cuando	Nunca	NS (no leer)	NC (no leer)
Me he sentido alegre y de buen humor	○	○	○	○	○	○	○	○
Me he sentido tranquilo y relajado	○	○	○	○	○	○	○	○
Me he sentido activo y enérgico	○	○	○	○	○	○	○	○
Me he despertado fresco y descansado	○	○	○	○	○	○	○	○
Mi vida cotidiana ha estado llena de cosas que me interesan	○	○	○	○	○	○	○	○

206

73. TABACO
De las situaciones siguientes, ¿cual describe mejor su comportamiento respecto al tabaco? (no leer: incluye cigarrillos, cigarros y pipas) *

- ○ Actualmente no fuma nada
- ○ Actualmente fuma ocasionalmente (menos de una vez al día)
- ○ Actualmente fuma cada día
- ○ NS (no leer)
- ○ NC (no leer)

207

74. ALCOHOL

Durante los últimos doce meses, ¿Con qué frecuencia ha consumido bebidas que contengan alcohol (vino, sidra, cerveza, brandy...)? *

- Diariamente
- De 4 a 6 veces por semana
- De 2 a 3 veces por semana
- Una vez por semana
- Una vez cada quince días
- Una vez al mes
- Menos de alguna vez al mes
- No he bebido en los últimos 12 meses
- NS (no leer)
- NC (no leer)

389

75. Durante los últimos 7 días, cuantos días ha realizado alguna actividad física o deportiva vigorosa o moderada en el TIEMPO LIBRE durante al menos 10 minutos seguidos? Ejemplos: fútbol, basquet, montañismo, atletismo, ciclismo, natación de competición, ir en bici, aeróbic, correr, tenis, natación, baile, yoga... *

- Días
- NS (no leer)
- NC (no leer)

ID 440

76. De la lista siguiente, qué medicamentos ha tomado durante el último mes? *

	Sí	No	NS (no leer)	NC (no leer)
Tranquilizantes, sedantes	○	○	○	○
Antidepresivos	○	○	○	○
Corticoesteroides	○	○	○	○
Anticonceptivos (només dones)	○	○	○	○
Medicamentos para dormir	○	○	○	○
Otro medicamento	○	○	○	○

LOGIC Hidden unless: Question "Otro medicamento" is one of the following answers ("Sí")

ID 521

77. ¿Cuál?

Redes sociales

Page description:
APOYO

ID 228

78. Ha acudido alguna vez en el último mes a alguna ONG, movimiento social o entiddad privada para solicitar algun tipo de apoyo económico o material (Cáritas, Cruz Roja, PAH, banco de alimentos, asociaciones...)?

*

- ○ Sí
- ○ No
- ○ NS (No leer)
- ○ NC (no leer)

ID 450

79. Participa o colabora con alguna de las asociaciones o organizaciones de voluntariado siguientes? *

	Sí	No	NS (no leer)	NC (no leer)
Club o asociación deportiva	○	○	○	○
Grupo cultural o de ocio	○	○	○	○
Asociación o grupo religioso	○	○	○	○
Asociación de vecinos	○	○	○	○
Partido o organización política	○	○	○	○
Sindicato	○	○	○	○
Asociación escolar o educativa	○	○	○	○
ONGs (Aministia Int, Oxfam, ACNUR, Creu Roja, Caritas, Unicef...)	○	○	○	○
Otras (cualquier grupo o organitzación feminista, ecologista, animalista...) Leer solo en caso de duda	○	○	○	○

LOGIC Hidden unless: Question "Otras (cualquier grupo o organitzación feminista, ecologista, animalista...) Leer solo en caso de duda" is one of the following answers ("Sí")

ID 246

¿Cuál?

[ ]

LOGIC Hidden unless: ((((((((Question "Club o asociación deportiva" is one of the following answers ("Sí") OR Question "Grupo cultural o de ocio" is one of the following answers ("Sí")) OR Question "Asociación o grupo religioso" is one of the following answers ("Sí")) OR Question "Asociación de vecinos" is one of the following answers ("Sí")) OR Question "Partido o organización política" is one of the following answers ("Sí")) OR Question "Sindicato" is one of the following answers ("Sí")) OR Question "Asociación escolar o educativa" is one of the following answers ("Sí")) OR Question "ONGs (Aministia Int, Oxfam, ACNUR, Creu Roja, Caritas, Unicef...)" is one of the following answers ("Sí")) OR Question "Otras (cualquier grupo o organitzación feminista, ecologista, animalista...) Leer solo en caso de duda" is one of the following answers ("Sí"))

ID 247

80. En caso que sí:
¿De qué manera participa o colabora (en la que participa más o considera más importante)? *

- Únicamente con una colaboración económica (pagar cuotas, donaciones, etc)
- Participación esporádica o puntual
- Participación como miembro activo

ID 248

81. ¿Existe en su empresa u organización un sindicato, comité de empresa o un comité similar que represente a los empleados? *

- Si
- No
- NS (no leer)
- NC (no leer)

ID 249

82. En el último mes, ¿ha participado en alguna reunión o asamblea de trabajadores de su empresa? *

- Si
- No
- NS (no leer)
- NC (no leer)

210

83. A continuación le leeré unas frases relacionadas con su vida social y el apoyo afectivo que recibe de otras personas. Valore si tiene tanto como desearía (5) o mucho menos de lo que desearía (1).
MOSTRAR TARJETA **13** *

	5	4	3	2	1	NS (no leer)	NC (no leer)
Recibe visitas de sus familiares y amigos	○	○	○	○	○	○	○
Recibe ayuda en temas relacionados con su casa	○	○	○	○	○	○	○
Recibe elogios y reconocimientos cuando hace bien su trabajo	○	○	○	○	○	○	○
Tiene personas que se preocupan del que le pasa	○	○	○	○	○	○	○
Recibe amor y afecto	○	○	○	○	○	○	○
Tiene la posibilidad de hablar con alguien de sus problemas en el trabajo y en el hogar	○	○	○	○	○	○	○
Tiene la posibilidad de hablar con alguien de sus problemas personales y familiares	○	○	○	○	○	○	○
Tiene la posibilidad de hablar con alguien de sus problemas económicos	○	○	○	○	○	○	○
Tiene invitaciones para distraerse y salir con otras personas	○	○	○	○	○	○	○
Recibe consejos útiles cuando le pasa alguna cosa importante a a la vida	○	○	○	○	○	○	○
Recibe ayuda cuando está enfermo/a en la cama	○	○	○	○	○	○	○

222

84. De la siguiente lista de afirmaciones, dígame si pueden ser ciertas o no en su caso:

MOSTRAR TARJETA **14** *

	Completamente cierto	Probablemente cierto	Probablemente falso	Completamente falso	NS (no leer)	NC (no leer)

Si necesitara un lugar para estar durante una semana por una emergencia (por ejemplo, un corte de agua o electricidad en mi apartamento o casa) podría fácilmente encontrar a alguien que me acogiera.	○	○	○	○	○	○
Hay alguien a quien podría acudir para pedir consejo acerca de mis planes sobre la carrera o cambios en el trabajo.	○	○	○	○	○	○
Sería difícil para mí encontrar a alguien que me prestara su coche durante unas horas	○	○	○	○	○	○
Si necesitara un préstamo de emergencia de 300 euros, hay alguien (amigo [illegible]	○	○	○	○	○	○

(amigo, familiar o conocido) que me lo daría.				

Condiciones de vida

Page description:
RIESGO DE POBREZA

LOGIC Show/hide trigger exists.

ID 251

85. Actualmente, ¿cuál es su situación de convivencia, es decir, usted vive ...? MOSTRAR TARJETA **15** *

- Con su pareja/cónyuge (solos)
- Con su pareja/cónyuge e hijos/as
- Con su pareja/cónyuge, hijos/as y otras personas (ascendientes, parientes...)
- Con su pareja/cónyuge y otras personas (ascendientes, parientes...)
- Solo/a
- Solo/a con sus hijos/as
- Solo/a con sus hijos/as y otras personas (ascendiente/parientes)
- Con sus padres (padre y/o madre)
- Con sus padres (padre y/o madre) y hermanos/as
- Con sus padres (padre i/o madre), hermanos/as y otras personas (ascendientes/parientes)
- Con sus padres (padre i/o madre) y otras personas (ascendientes/parientes)
- Con su hijo/a y nietos
- Con otros familiares
- Con amigos/as o compañeros/as de piso
- Otras situaciones. Especifíquela
- NS (no leer)
- NC (no leer)

VALIDATION Must be numeric

LOGIC Hidden unless: #85 Question "Actualmente, ¿cuál es su situación de convivencia, es decir, usted vive ...?
MOSTRAR TARJETA **15**" is one of the following answers ("Con su pareja/cónyuge (solos)","Con su pareja/cónyuge e hijos/as","Con su pareja/cónyuge, hijos/as y otras personas (ascendientes, parientes...)","Con su pareja/cónyuge y otras personas (ascendientes, parientes...)","Solo/a con sus hijos/as","Solo/a con sus hijos/as y otras personas (ascendiente/parientes)","Con sus padres (padre y/o madre)","Con sus padres (padre y/o madre) y hermanos/as","Con sus padres (padre i/o madre), hermanos/as y otras personas (ascendientes/parientes)","Con sus padres (padre i/o madre) y otras personas (ascendientes/parientes)","Con su hijo/a y nietos","Con otros familiares","Con amigos/as o compañeros/as de piso","Otras situaciones. Especifíquela","NS (no leer)","NC (no leer)")

ID 252

86. Cuántas personas residen en esta vivienda contándose a usted? (no es necesario estar empadronado?

Leer estos puntos en caso de duda de la persona:

1) son residentes las personas que duermen allí normalmente
2) las personas que ahora son fuera por cuestiones relacionadas con los estudios, trabajo o otras cuestiones se cuentan como residentes en el hogar si tienen previsto volver en menos de un año.
3) Si hay hijos de padres separados, se considera que residen en este hogar si es en el que pasan más tiempo.
4) Si hay hijos de padres separados que pasan el mismo tiempo en los dos domicilios se considera que residen en el hogar si duermen el día en que se contesta la encuesta
5) Los asistentes del hogar y el personal doméstico es considerado que viven en este hogar solo si duermen normalmente cuando no están trabajando
6) Si se tiene más de un hogar se reside en este hogar solo si es en el que pasa más tiempo teniendo en cuenta todo el año
7) Los estudiantes menores de edad que viven fuera durante el curso o los días laborales cuentan como residentes en el hogar. *

VALIDATION Must be numeric

LOGIC Hidden unless: #85 Question "Actualmente, ¿cuál es su situación de convivencia, es decir, usted vive ...?
MOSTRAR TARJETA **15**" is one of the following answers ("Con su pareja/cónyuge (solos)","Con su pareja/cónyuge e hijos/as","Con su pareja/cónyuge, hijos/as y otras personas (ascendientes, parientes...)","Con su pareja/cónyuge y otras personas (ascendientes, parientes...)","Solo/a con sus hijos/as","Solo/a con sus hijos/as y otras personas (ascendiente/parientes)","Con sus padres (padre y/o madre)","Con sus padres (padre y/o madre) y hermanos/as","Con sus padres (padre i/o madre), hermanos/as y otras personas (ascendientes/parientes)","Con sus padres (padre i/o madre) y otras personas (ascendientes/parientes)","Con su hijo/a y nietos","Con otros familiares","Con amigos/as o compañeros/as de piso","Otras situaciones. Especifíquela","NS (no leer)","NC (no leer)")

ID 253

87. ¿Con cuantas personas de las que convive en este piso o casa comparte los gastos principales relacionados con el hogar y las alimentación?(contándose a usted). *

LOGIC Show/hide trigger exists.

ID 259

88. Por lo tanto, en su hogar conviven (contandose a usted):
* hace referencia a la última pregunta, de personas con las que convive y comparte gastos
* hijos de 14 años o más cuentan como adultos
*

- 1 adult (hogar unipersonal)
- 2 adultos
- 3 adultos
- 4 adultos
- 5 adultos
- 6 adultos
- 7 adultos
- 8 adultos
- 1 adulto y 1 niño
- 1 adulto y 2 niños
- 1 adulto y 3 niños

- 2 adultos y 1 niño
- 2 adultos y 2 niños
- 2 adultos y 3 niños
- 3 adultos y 1 niño
- 3 adultos y 2 niños
- 3 adultos y 3 niños
- 4 adultos y 1 niño
- 4 adultos y 2 niños
- 4 adultos y 3 niños
- 5 adultos y 1 niño
- 5 adultos y 2 niños
- 5 adultos y 3 niños
- Otros
- NS (no leer)
- NC (no leer)

VALIDATION Must be numeric

ID 470

89. **Persona 1 (entrevistado/a):**

Durante los últimos 12 meses, ¿Cuántos meses trabajó, aunque fuese solamente unas horas?

(0 en caso de que no trabaje, no preguntarlo en menores 16)

VALIDATION Must be numeric

ID 471

90. **Persona 1 (entrevistado/a):**

¿Cuántas horas semanales trabajó habitualmente durante los últimos 12 meses?

(Si pregunta anterior = 0, no preguntarlo y anotar directamente 0)

(Si era asalariado, incluya las horas extras que realizó habitualmente. Si el trabajo era irregular indique la media de horas semanales trabajadas. Contar sólo los meses en los que estuvo trabajando).

VALIDATION Must be numeric

LOGIC Show/hide trigger exists. Hidden unless: #88 Question "Por lo tanto, en su hogar conviven (contandose a usted):

* hace referencia a la última pregunta, de personas con las que convive y comparte gastos
* hijos de 14 años o más cuentan como adultos

" is one of the following answers ("2 adultos","3 adultos","4 adultos","5 adultos","6 adultos","7 adultos","8 adultos","1 adulto y 1 niño","1 adulto y 2 niños","1 adulto y 3 niños","2 adultos y 1 niño","2 adultos y 2 niños","2 adultos y 3 niños","3 adultos y 1 niño","3 adultos y 2 niños","3 adultos y 3 niños","4 adultos y 1 niño","4 adultos y 2 niños","4 adultos y 3 niños","5 adultos y 1 niño","5 adultos y 2 niños","5 adultos y 3 niños","Otros")

ID 477

91. **Persona 2**: Edad *

VALIDATION Must be numeric

LOGIC Hidden unless: #91 Question "**Persona 2**: Edad" is greater than or equal to "16"

ID 474

92. **Persona 2:**

Durante los últimos 12 meses, ¿Cuántos meses trabajó, aunque fuese solamente unas horas?

(0 en caso de que no trebaje)

(Menores de 16 años: no preguntarlo y anotar directamente 0)

VALIDATION Must be numeric

LOGIC Hidden unless: (#91 Question "**Persona 2**: Edad" is greater than or equal to "16" AND #92 Question "**Persona 2:**
Durante los últimos 12 meses, ¿Cuántos meses trabajó, aunque fuese solamente unas horas?
(0 en caso de que no trebaje)
(Menores de 16 años: no preguntarlo y anotar directamente 0)" is greater than "0")

ID 475

93. **Persona 2:**
¿Cuántas horas semanales trabajó habitualmente durante los últimos 12 meses?
(Si pregunta anterior = 0, no preguntarlo y anotar directamente 0)
(Si era asalariado, incluya las horas extras que realizó habitualmente. Si el trabajo era irregular indique la media de horas semanales trabajadas. Contar sólo los meses en los que estuvo trabajando).

VALIDATION Must be numeric

LOGIC Show/hide trigger exists. Hidden unless: #88 Question "Por lo tanto, en su hogar conviven (contandose a usted):
* hace referencia a la última pregunta, de personas con las que convive y comparte gastos
* hijos de 14 años o más cuentan como adultos
" is one of the following answers ("3 adultos","4 adultos","5 adultos","6 adultos","7 adultos","8 adultos","1 adulto y 2 niños","1 adulto y 3 niños","2 adultos y 1 niño","2 adultos y 2 niños","2 adultos y 3 niños","3 adultos y 1 niño","3 adultos y 2 niños","3 adultos y 3 niños","4 adultos y 1 niño","4 adultos y 2 niños","4 adultos y 3 niños","5 adultos y 1 niño","5 adultos y 2 niños","5 adultos y 3 niños","Otros")

ID 484

94. **Persona 3**: Edad *

VALIDATION Must be numeric

LOGIC Hidden unless: #94 Question "**Persona 3**: Edad" is greater than or equal to "16"

ID 499

95. **Persona 3:**

Durante los últimos 12 meses, cuántos meses trabajó, aunque fuese solamente unas horas?

(0 en caso de que no trebaje)

(Menores de 16 años: no preguntarlo y anotar directamente 0)

VALIDATION Must be numeric

LOGIC Hidden unless: (#94 Question "**Persona 3**: Edad" is greater than or equal to "16" AND #95 Question "**Persona 3:**
Durante los últimos 12 meses, cuántos meses trabajó, aunque fuese solamente unas horas?
(0 en caso de que no trebaje)
(Menores de 16 años: no preguntarlo y anotar directamente 0)" is greater than "0")

ID 493

96. **Persona 3:**

¿Cuántas horas semanales trabajó habitualmente durante los últimos 12 meses?

(Si pregunta anterior = 0, no preguntarlo y anotar directamente 0)

(Si era asalariado, incluya las horas extras que realizó habitualmente. Si el trabajo era irregular indique la media de horas semanales trabajadas. Contar sólo los meses en los que estuvo trabajando).

VALIDATION Must be numeric

LOGIC Show/hide trigger exists. Hidden unless: #88 Question "Por lo tanto, en su hogar conviven (contandose a usted):
* hace referencia a la última pregunta, de personas con las que convive y comparte gastos
* hijos de 14 años o más cuentan como adultos
" is one of the following answers ("4 adultos","5 adultos","6 adultos","7 adultos","8 adultos","1 adulto y 3 niños","2 adultos y 2 niños","2 adultos y 3 niños","3 adultos y 1 niño","3 adultos y 2 niños","3 adultos y 3 niños","4 adultos y 1 niño","4 adultos y 2 niños","4 adultos y 3 niños","5 adultos y 1 niño","5 adultos y 2 niños","5 adultos y 3 niños","Otros")

ID 485

97. **Persona 4**: Edad *

VALIDATION Must be numeric

LOGIC Hidden unless: #97 Question "**Persona 4**: Edad" is greater than or equal to "16"

ID 504

98. **Persona 4:**

Durante los últimos 12 meses, cuántos meses trabajó, aunque fuese solamente unas horas?

(0 en caso de que no trebaje)

(Menores de 16 años: no preguntarlo y anotar directamente 0)

VALIDATION Must be numeric

LOGIC Hidden unless: (#97 Question "**Persona 4**: Edad" is greater than or equal to "16" AND #98 Question "**Persona 4:**
Durante los últimos 12 meses, cuántos meses trabajó, aunque fuese solamente unas horas?
(0 en caso de que no trebaje)
(Menores de 16 años: no preguntarlo y anotar directamente 0)" is greater than "0")

ID 495

99. **Persona 4:**
¿Cuántas horas semanales trabajó habitualmente durante los últimos 12 meses?
(Si pregunta anterior = 0, no preguntarlo y anotar directamente 0)
(Si era asalariado, incluya las horas extras que realizó habitualmente. Si el trabajo era irregular indique la media de horas semanales trabajadas. Contar sólo los meses en los que estuvo trabajando).

VALIDATION Must be numeric

LOGIC Show/hide trigger exists. Hidden unless: #88 Question "Por lo tanto, en su hogar conviven (contandose a usted):
* hace referencia a la última pregunta, de personas con las que convive y comparte gastos
* hijos de 14 años o más cuentan como adultos
" is one of the following answers ("5 adultos","6 adultos","7 adultos","8 adultos","2 adultos y 3 niños","3 adultos y 2 niños","3 adultos y 3 niños","4 adultos y 1 niño","4 adultos y 2 niños","4 adultos y 3 niños","5 adultos y 1 niño","5 adultos y 2 niños","5 adultos y 3 niños","Otros")

ID 489

100. **Persona 5**: Edad *

VALIDATION Must be numeric

LOGIC Hidden unless: #100 Question "**Persona 5**: Edad" is greater than or equal to "16"

ID 503

101. **Persona 5:**
Durante los últimos 12 meses, cuántos meses trabajó, aunque fuese solamente unas horas?
(0 en caso de que no trebaje)
(Menores de 16 años: no preguntarlo y anotar directamente 0)

VALIDATION Must be numeric

LOGIC Hidden unless: (#100 Question "**Persona 5**: Edad" is greater than or equal to "16" AND #101 Question "**Persona 5:**
Durante los últimos 12 meses, cuántos meses trabajó, aunque fuese solamente unas horas?
(0 en caso de que no trebaje)
(Menores de 16 años: no preguntarlo y anotar directamente 0)" is greater than "0")

ID 496

102. **Persona 5:**
¿Cuántas horas semanales trabajó habitualmente durante los últimos 12 meses?
(Si pregunta anterior = 0, no preguntarlo y anotar directamente 0)
(Si era asalariado, incluya las horas extras que realizó habitualmente. Si el trabajo era irregular indique la media de horas semanales trabajadas. Contar sólo los meses en los que estuvo trabajando).

VALIDATION Must be numeric

LOGIC Show/hide trigger exists. Hidden unless: #88 Question "Por lo tanto, en su hogar conviven (contandose a usted):
* hace referencia a la última pregunta, de personas con las que convive y comparte gastos
* hijos de 14 años o más cuentan como adultos
" is one of the following answers ("6 adultos","7 adultos","8 adultos","3 adultos y 3 niños","4 adultos y 2 niños","4 adultos y 3 niños","5 adultos y 1 niño","5 adultos y 2 niños","5 adultos y 3 niños","Otros")

ID 490

103. **Persona 6**: Edad *

VALIDATION Must be numeric

LOGIC Hidden unless: #103 Question "**Persona 6**: Edad" is greater than or equal to "16"

ID 502

104. **Persona 6:**
Durante los últimos 12 meses, cuántos meses trabajó, aunque fuese solamente unas horas?
(0 en caso de que no trebaje)
(Menores de 16 años: no preguntarlo y anotar directamente 0)

VALIDATION Must be numeric

LOGIC Hidden unless: (#103 Question "**Persona 6**: Edad" is greater than or equal to "16" AND #104 Question "**Persona 6:**
Durante los últimos 12 meses, cuántos meses trabajó, aunque fuese solamente unas horas?
(0 en caso de que no trebaje)
(Menores de 16 años: no preguntarlo y anotar directamente 0)" is greater than "0")

ID 494

105. **Persona 6:**
¿Cuántas horas semanales trabajó habitualmente durante los últimos 12 meses?
(Si pregunta anterior = 0, no preguntarlo y anotar directamente 0)
(Si era asalariado, incluya las horas extras que realizó habitualmente. Si el trabajo era irregular indique la media de horas semanales trabajadas. Contar sólo los meses en los que estuvo trabajando).

VALIDATION Must be numeric

LOGIC Show/hide trigger exists. Hidden unless: #88 Question "Por lo tanto, en su hogar conviven (contandose a usted):
* hace referencia a la última pregunta, de personas con las que convive y comparte gastos
* hijos de 14 años o más cuentan como adultos
" is one of the following answers ("7 adultos","8 adultos","4 adultos y 3 niños","5 adultos y 2 niños","5 adultos y 3 niños","Otros")

ID 491

106. **Persona 7**: Edad *

VALIDATION Must be numeric

LOGIC Hidden unless: #106 Question "**Persona 7**: Edad" is greater than or equal to "16"

ID 501

107. **Persona 7:**
Durante los últimos 12 meses, cuántos meses trabajó, aunque fuese solamente unas horas?
(0 en caso de que no trebaje)
(Menores de 16 años: no preguntarlo y anotar directamente 0)

VALIDATION Must be numeric

LOGIC Hidden unless: (#106 Question "**Persona 7**: Edad" is greater than or equal to "16" AND #107 Question "**Persona 7:**
Durante los últimos 12 meses, cuántos meses trabajó, aunque fuese solamente unas horas?
(0 en caso de que no trebaje)
(Menores de 16 años: no preguntarlo y anotar directamente 0)" is greater than "0")

ID 497

108. **Persona 7:**
¿Cuántas horas semanales trabajó habitualmente durante los últimos 12 meses?
(Si pregunta anterior = 0, no preguntarlo y anotar directamente 0)
(Si era asalariado, incluya las horas extras que realizó habitualmente. Si el trabajo era irregular indique la media de horas semanales trabajadas. Contar sólo los meses en los que estuvo trabajando).

VALIDATION Must be numeric

LOGIC Show/hide trigger exists. Hidden unless: #88 Question "Por lo tanto, en su hogar conviven (contandose a usted):
* hace referencia a la última pregunta, de personas con las que convive y comparte gastos
* hijos de 14 años o más cuentan como adultos
" is one of the following answers ("8 adultos","5 adultos y 3 niños","Otros")

ID 492

109. **Persona 8**: Edad *

VALIDATION Must be numeric

LOGIC Hidden unless: #109 Question "**Persona 8**: Edad" is greater than or equal to "16"

ID 500

110. **Persona 8:**
Durante los últimos 12 meses, cuántos meses trabajó, aunque fuese solamente unas horas?
(0 en caso de que no trebaje)
(Menores de 16 años: no preguntarlo y anotar directamente 0)

VALIDATION Must be numeric

LOGIC Hidden unless: (#109 Question "**Persona 8**: Edad" is greater than or equal to "16" AND #110 Question "**Persona 8:**
Durante los últimos 12 meses, cuántos meses trabajó, aunque fuese solamente unas horas?
(0 en caso de que no trebaje)
(Menores de 16 años: no preguntarlo y anotar directamente 0)" is greater than "0")

ID 498

111. **Persona 8:**
¿Cuántas horas semanales trabajó habitualmente durante los últimos 12 meses?
(Si pregunta anterior = 0, no preguntarlo y anotar directamente 0)
(Si era asalariado, incluya las horas extras que realizó habitualmente. Si el trabajo era irregular indique la media de horas semanales trabajadas. Contar sólo los meses en los que estuvo trabajando).

VALIDATION Must be numeric

LOGIC Show/hide trigger exists. Hidden unless: #88 Question "Por lo tanto, en su hogar conviven (contandose a usted):
* hace referencia a la última pregunta, de personas con las que convive y comparte gastos
* hijos de 14 años o más cuentan como adultos
" is one of the following answers ("Otros")

ID 507

112. **Persona 9**: Edad *

VALIDATION Must be numeric

LOGIC Hidden unless: #112 Question "**Persona 9**: Edad" is greater than or equal to "16"

ID 509

113. **Persona 9:**
Durante los últimos 12 meses, cuántos meses trabajó, aunque fuese solamente unas horas?
(0 en caso de que no trebaje)
(Menores de 16 años: no preguntarlo y anotar directamente 0)

VALIDATION Must be numeric

LOGIC Hidden unless: (#112 Question "**Persona 9**: Edad" is greater than or equal to "16" AND #113 Question "**Persona 9:**
Durante los últimos 12 meses, cuántos meses trabajó, aunque fuese solamente unas horas?
(0 en caso de que no trebaje)
(Menores de 16 años: no preguntarlo y anotar directamente 0)" is greater than "0")

ID 511

114. **Persona 9:**

¿Cuántas horas semanales trabajó habitualmente durante los últimos 12 meses?

(Si pregunta anterior = 0, no preguntarlo y anotar directamente 0)

(Si era asalariado, incluya las horas extras que realizó habitualmente. Si el trabajo era irregular indique la media de horas semanales trabajadas. Contar sólo los meses en los que estuvo trabajando).

VALIDATION Must be numeric

LOGIC Show/hide trigger exists. Hidden unless: #88 Question "Por lo tanto, en su hogar conviven (contandose a usted):
* hace referencia a la última pregunta, de personas con las que convive y comparte gastos
* hijos de 14 años o más cuentan como adultos
" is one of the following answers ("Otros")

ID 508

115. **Persona 10**: Edad *

VALIDATION Must be numeric

LOGIC Hidden unless: #115 Question "**Persona 10**: Edad" is greater than or equal to "16"

ID 510

116. **Persona 10:**

Durante los últimos 12 meses, cuántos meses trabajó, aunque fuese solamente unas horas?

(0 en caso de que no trebaje)

(Menores de 16 años: no preguntarlo y anotar directamente 0)

VALIDATION Must be numeric

LOGIC Hidden unless: (#115 Question "**Persona 10**: Edad" is greater than or equal to "16" AND #116 Question "**Persona 10:**
Durante los últimos 12 meses, cuántos meses trabajó, aunque fuese solamente unas horas?
(0 en caso de que no trebaje)
(Menores de 16 años: no preguntarlo y anotar directamente 0)" is greater than "0")

ID 512

117. **Persona 10:**
¿Cuántas horas semanales trabajó habitualmente durante los últimos 12 meses?
(Si pregunta anterior = 0, no preguntarlo y anotar directamente 0)
(Si era asalariado, incluya las horas extras que realizó habitualmente. Si el trabajo era irregular indique la media de horas semanales trabajadas. Contar sólo los meses en los que estuvo trabajando).

LOGIC Show/hide trigger exists.

ID 528

118. De las personas que viven en su hogar, alguna necesita de su cuidado de manera habitual (hijos menores de 15 años, personas discapacitadas, personas mayores no autónomas...)?

- Si
- No
- NS (no leer)
- NC (no leer)

LOGIC Hidden unless: #118 Question "De las personas que viven en su hogar, alguna necesita de su cuidado de manera habitual (hijos menores de 15 años, personas discapacitadas, personas mayores no autónomas...)?" is one of the following answers ("Si")

ID 529

119. ¿Cuantas horas aproximadamente de media dedica al día a la tarea de cuidados de esta persona dependiente?

LOGIC Hidden unless: #88 Question "Por lo tanto, en su hogar conviven (contandose a usted):
* hace referencia a la última pregunta, de personas con las que convive y comparte gastos
* hijos de 14 años o más cuentan como adultos
" is one of the following answers ("2 adultos","3 adultos","4 adultos","5 adultos","6 adultos","7 adultos","8 adultos","1 adulto y 1 niño","1 adulto y 2 niños","1 adulto y 3 niños","2 adultos y 1 niño","2 adultos y 2 niños","2 adultos y 3 niños","3 adultos y 1 niño","3 adultos y 2 niños","3 adultos y 3 niños","4 adultos y 1 niño","4 adultos y 2 niños","4 adultos y 3 niños","5 adultos y 1 niño","5 adultos y 2 niños","5 adultos y 3 niños","Otros")

ID 260

120. Me podría indicar el importe de los ingresos (netos) mensuales medios del hogar teniendo en cuenta todos sus miembros?

Tenga en cuenta lo siguiente:
- Tener en cuenta los ingresos de todos los miembros del hogar
- Estamos pidiendo ingresos netos (una vez realizadas las retenciones, cotizaciones sociales y pagados los impuestos pertinentes)
- Si los ingresos varían de un mes a otro, haga una aproximación a la media de los últimos 12 meses
- Tenga en cuenta los siguientes ingresos: salario, ingresos por actividades profesionales o empresariales, prestaciones*, ayudas, alquiler de propiedades, intereses, dividendos, ganancias y otros ingresos financieros y transferencias recibidas de otras familias.

*Desempleo o formación profesional; pensiones o planes privados de jubilación o vejez, viudedad, orfandad o otras prestaciones por supervivencia, protección a la familia (maternidad, cura de hijos o personas mayores), enfermedad, invalidez, ayudas escolares o becas, ayudas de asistencia social, ayudas a la vivienda. *

. *

- ○ Cantidad neta mensual ingresada
- ○ No lo sé (no leer)
- ○ NC (no leer)

LOGIC Show/hide trigger exists. Hidden unless: #88 Question "Por lo tanto, en su hogar conviven (contandose a usted):
* hace referencia a la última pregunta, de personas con las que convive y comparte gastos
* hijos de 14 años o más cuentan como adultos
" is one of the following answers ("1 adult (hogar unipersonal)")

ID 468

121. Me podría indicar el importe de sus ingresos (netos) mensuales medios totales?

Tenga en cuenta lo siguiente:
- Tener en cuenta los ingresos de todos los miembros del hogar
- Estamos pidiendo ingresos netos (una vez realizadas las retenciones, cotizaciones sociales y pagados los impuestos pertinentes)
- Si los ingresos varían de un mes a otro, haga una aproximación a la media de los últimos 12 meses
- Tenga en cuenta los siguientes ingresos: salario, ingresos por actividades profesionales o empresariales, prestaciones*, ayudas, alquiler de propiedades, intereses, dividendos, ganancias y otros ingresos financieros y transferencias recibidas de otras familias.

*Desempleo o formación profesional; pensiones o planes privados de jubilación o vejez, viudedad, orfandad o otras prestaciones por supervivencia, protección a la familia (maternidad, cura de hijos o personas mayores), enfermedad, invalidez, ayudas escolares o becas, ayudas de asistencia social, ayudas a la vivienda. *

- ( ) Cantidad neta mensual ingresada [ ]
- ( ) No lo se
- ( ) Prefiero con contestar

LOGIC Hidden unless: (#88 Question "Por lo tanto, en su hogar conviven (contandose a usted):
* hace referencia a la última pregunta, de personas con las que convive y comparte gastos
* hijos de 14 años o más cuentan como adultos
" is one of the following answers ("2 adultos","3 adultos","4 adultos","5 adultos","6 adultos","7 adultos","8 adultos","1 adulto y 1 niño","1 adulto y 2 niños","1 adulto y 3 niños","2 adultos y 1 niño","2 adultos y 2 niños","2 adultos y 3 niños","3 adultos y 1 niño","3 adultos y 2 niños","3 adultos y 3 niños","4 adultos y 1 niño","4 adultos y 2 niños","4 adultos y 3 niños","5 adultos y 1 niño","5 adultos y 2 niños","5 adultos y 3 niños") AND Question "." is one of the following answers ("No lo sé (no leer)","NC (no leer)"))

ID 264

122. (Si el hogar está formado por más de una persona) Podría decir-nos aproximadamente en qué tramo se situarían los ingresos totales de su hogar MOSTRAR TARJETA **16**

*

- 1) 500 euros al mes, más o menos
- 2) Entre 501 y 1000 euros al mes
- 3) Entre 1001 y 1500 euros al mes
- 4) Entre 1501 y 2000 euros al mes
- 5) Entre 2001 y 2500 euros al mes
- 6) Entre 2501 y 3000 euros al mes
- 7) Entre 3001 y 3500 euros al mes
- 8) Entre 3501 y 4000 euros al mes
- 9) Entre 4001 y 4500 euros al mes
- 10) Entre 4501 y 5000 euros al mes
- 11) Entre 5001 y 5500 euros al mes
- 12) Entre 5501 y 6000 euros al mes
- 13) Más de 6000 euros al mes
- NS (no leer)
- NC (no leer)

LOGIC Hidden unless: #121 Question "Me podría indicar el importe de sus ingresos (netos) mensuales medios totales?

Tenga en cuenta lo siguiente:

- Tener en cuenta los ingresos de todos los miembros del hogar
- Estamos pidiendo ingresos netos (una vez realizadas las retenciones, cotizaciones sociales y pagados los impuestos pertinentes)
- Si los ingresos varían de un mes a otro, haga una aproximación a la media de los últimos 12 meses
- Tenga en cuenta los siguientes ingresos: salario, ingresos por actividades profesionales o empresariales, prestaciones*, ayudas, alquiler de propiedades, intereses, dividendos, ganancias y otros ingresos financieros y transferencias recibidas de otras familias.

*Desempleo o formación profesional; pensiones o planes privados de jubilación o vejez, viudedad, orfandad o otras prestaciones por supervivencia, protección a la familia (maternidad, cura de hijos o personas mayores), enfermedad, invalidez, ayudas escolares o becas, ayudas de asistencia social, ayudas a la vivienda." is one of the following answers ("No lo se","Prefiero con contestar")

ID 362

123. (Si el hogar está formado por una persona) Podría decirnos aproximadamente en qué tramo se sitúan sus ingresos totales?

MOSTRAR TARJETA **16**

*

- 1) 500 euros al mes, más o menos
- 2) Entre 501 y 1000 euros al mes
- 3) Entre 1001 y 1500 euros al mes
- 4) Entre 1501 y 2000 euros al mes
- 5) ntre 2001 y 2500 euros al mes
- 6) Entre 2501 y 3000 euros al mes
- 7) Entre 3001 y 3500 euros al mes
- 8) Entre 3501 y 4000 euros al mes
- 9) Entre 4001 y 4500 euros al mes
- 10) Entre 4501 y 5000 euros al mes
- 11) Entre 5001 y 5500 euros al mes
- 12) Entre 5501 y 6000 euros al mes
- 13) Más de 6000 euros al mes
- NS (no leer)
- NC (no leer)

LOGIC Hidden unless: (#88 Question "Por lo tanto, en su hogar conviven (contandose a usted):
* hace referencia a la última pregunta, de personas con las que convive y comparte gastos
* hijos de 14 años o más cuentan como adultos
" is one of the following answers ("1 adult (hogar unipersonal)") AND #121 Question "Me podría indicar el importe de sus ingresos (netos) mensuales medios totales?

Tenga en cuenta lo siguiente:
- Tener en cuenta los ingresos de todos los miembros del hogar
- Estamos pidiendo ingresos netos (una vez realizadas las retenciones, cotizaciones sociales y pagados los impuestos pertinentes)
- Si los ingresos varían de un mes a otro, haga una aproximación a la media de los últimos 12 meses
- Tenga en cuenta los siguientes ingresos: salario, ingresos por actividades profesionales o empresariales, prestaciones*, ayudas, alquiler de propiedades, intereses, dividendos, ganancias y otros ingresos financieros y transferencias recibidas de otras familias.

*Desempleo o formación profesional; pensiones o planes privados de jubilación o vejez, viudedad, orfandad o otras prestaciones por supervivencia, protección a la familia (maternidad, cura de hijos o personas mayores), enfermedad, invalidez, ayudas escolares o becas, ayudas de asistencia social, ayudas a la vivienda." is one of the following answers ("No lo se","Prefiero con contestar"))

ID 267

124. Indique, por favor, si de media sus ingresos mensuales son más altos o más bajos que la cifra que aparece a continuación: **926, 87 €** *

- Ingresan, al mes, más que esta cantidad
- Ingresan, al mes, aproximadamente esta cantidad (+-100€)
- Ingresan, al mes, menos que esta cantidad
- NS (no leer)
- NC (no leer)

LOGIC Hidden unless: (#88 Question "Por lo tanto, en su hogar conviven (contandose a usted):
* hace referencia a la última pregunta, de personas con las que convive y comparte gastos
* hijos de 14 años o más cuentan como adultos
" is one of the following answers ("2 adultos") AND Question "." is one of the following answers ("No lo sé (no leer)","NC (no leer)"))

ID 268

125. Indique, por favor, si de media sus ingresos mensuales son más altos o más bajos que la cifra que aparece a continuación: **1204,88€** *

- Ingresan, al mes, más que esta cantidad
- Ingresan, al mes, aproximadamente esta cantidad (+-100€)
- Ingresan, al mes, menos que esta cantidad
- NS (no leer)
- NC (no leer)

LOGIC Hidden unless: (#88 Question "Por lo tanto, en su hogar conviven (contandose a usted):
* hace referencia a la última pregunta, de personas con las que convive y comparte gastos
* hijos de 14 años o más cuentan como adultos
" is one of the following answers ("3 adultos") AND Question "." is one of the following answers ("No lo sé (no leer)","NC (no leer)"))

ID 269

126. Indique, por favor, si de media sus ingresos mensuales son más altos o más bajos que la cifra que aparece a continuación: **1853,70€** *

- Ingresan, al mes, más que esta cantidad
- Ingresan, al mes, aproximadamente esta cantidad(+-100€)
- Ingresan, al mes, menos que esta cantidad
- NS (no llegir)
- NC (no llegir)

LOGIC Hidden unless: (#88 Question "Por lo tanto, en su hogar conviven (contandose a usted):
* hace referencia a la última pregunta, de personas con las que convive y comparte gastos
* hijos de 14 años o más cuentan como adultos
" is one of the following answers ("4 adultos") AND Question "." is one of the following answers ("No lo sé (no leer)","NC (no leer)"))

ID 270

127. Indique, por favor, si de media sus ingresos mensuales son más altos o más bajos que la cifra que aparece a continuación: **2317,20€** *

- Ingresan, al mes, más que esta cantidad
- Ingresan, al mes, aproximadamente aquesta cantidad (+-100€)
- Ingresan, al mes, menos que esta cantidad
- NS (no leer)
- NC (no leer)

LOGIC Hidden unless: (#88 Question "Por lo tanto, en su hogar conviven (contandose a usted):
* hace referencia a la última pregunta, de personas con las que convive y comparte gastos
* hijos de 14 años o más cuentan como adultos
" is one of the following answers ("5 adultos") AND Question "." is one of the following answers ("No lo sé (no leer)","NC (no leer)"))

ID 271

128. Indique, por favor, si de media sus ingresos mensuales son más altos o más bajos que la cifra que aparece a continuación: **2780,60€** *

- Ingresan, al mes, más que esta cantidad
- Ingresan, al mes, aproximadamente esta cantidad (+-100€)
- Ingresan, al mes, menos que esta cantidad
- NS (no leer)
- NC (no leer)

LOGIC Hidden unless: (#88 Question "Por lo tanto, en su hogar conviven (contandose a usted):
* hace referencia a la última pregunta, de personas con las que convive y comparte gastos
* hijos de 14 años o más cuentan como adultos
" is one of the following answers ("6 adultos") AND Question "." is one of the following answers ("No lo sé (no leer)","NC (no leer)"))

ID 363

129. Indique, por favor, si de media sus ingresos mensuales son más altos o más bajos que la cifra que aparece a continuación: **3244,0€** *

- Ingresan, al mes, más que esta cantidad
- Ingresan, al mes, aproximadamente esta cantidad (+-100€)
- Ingresan, al mes, menos que esta cantidad
- NS (no leer)
- NC (no leer)

LOGIC Hidden unless: (#88 Question "Por lo tanto, en su hogar conviven (contandose a usted):
* hace referencia a la última pregunta, de personas con las que convive y comparte gastos
* hijos de 14 años o más cuentan como adultos
" is one of the following answers ("7 adultos") AND Question "." is one of the following answers ("No lo sé (no leer)","NC (no leer)"))

ID 364

130. Indique, por favor, si de media sus ingresos mensuales son más altos o más bajos que la cifra que aparece a continuación: **3707,50€** *

- Ingresan, al mes, más que esta cantidad
- Ingresan, al mes, aproximadamente esta cantidad (+-100€)
- Ingresan, al mes, menos que esta cantidad
- NS (no leer)
- NC (no leer)

LOGIC Hidden unless: (#88 Question "Por lo tanto, en su hogar conviven (contandose a usted):
* hace referencia a la última pregunta, de personas con las que convive y comparte gastos
* hijos de 14 años o más cuentan como adultos
" is one of the following answers ("8 adultos") AND Question "." is one of the following answers ("No lo sé (no leer)","NC (no leer)"))

ID 365

131. Indique, por favor, si de media sus ingresos mensuales son más altos o más bajos que la cifra que aparece a continuación: **4170,90€** *

- Ingresan, al mes, más que esta cantidad
- Ingresan, al mes, aproximadamente esta cantidad(+-100€)
- Ingresan, al mes, menos que esta cantidad
- NS (no leer)
- NC (no leer)

LOGIC Hidden unless: (#88 Question "Por lo tanto, en su hogar conviven (contandose a usted):
* hace referencia a la última pregunta, de personas con las que convive y comparte gastos
* hijos de 14 años o más cuentan como adultos
" is one of the following answers ("1 adulto y 1 niño") AND Question "." is one of the following answers ("No lo sé (no leer)","NC (no leer)"))

ID 272

132. Indique, por favor, si de media sus ingresos mensuales son más altos o más bajos que la cifra que aparece a continuación: **1204,90€** *

- Ingresan, al mes, más que esta cantidad
- Ingresan, al mes, aproximadamente esta cantidad (+-100€)
- Ingresan, al mes, menos que esta cantidad
- NS (no leer)
- NC (no leer)

LOGIC Hidden unless: (#88 Question "Por lo tanto, en su hogar conviven (contandose a usted):
* hace referencia a la última pregunta, de personas con las que convive y comparte gastos
* hijos de 14 años o más cuentan como adultos
" is one of the following answers ("1 adulto y 2 niños") AND Question " " is one of the following answers ("No lo sé (no leer)","NC (no leer)"))

ID 273

133. Indique, por favor, si de media sus ingresos mensuales son más altos o más bajos que la cifra que aparece a continuación: **1483€** *

- Ingresan, al mes, más que esta cantidad
- Ingresan, al mes, aproximadamente esta cantidad (+-100€)
- Ingresan, al mes, menos que esta cantidad
- NS (no leer)
- NC (no leer)

LOGIC Hidden unless: (#88 Question "Por lo tanto, en su hogar conviven (contandose a usted):
* hace referencia a la última pregunta, de personas con las que convive y comparte gastos
* hijos de 14 años o más cuentan como adultos
" is one of the following answers ("1 adulto y 3 niños") AND Question " " is one of the following answers ("No lo sé (no leer)","NC (no leer)"))

ID 274

134. Indique, por favor, si de media sus ingresos mensuales son más altos o más bajos que la cifra que aparece a continuación: **1761€** *

- Ingresan, al mes, más que esta cantidad
- Ingresan, al mes, aproximadamente esta cantidad (+-100€)
- Ingresan, al mes, menos que esta cantidad
- NS (no leer)
- NC (no leer)

LOGIC Hidden unless: (#88 Question "Por lo tanto, en su hogar conviven (contandose a usted):
* hace referencia a la última pregunta, de personas con las que convive y comparte gastos
* hijos de 14 años o más cuentan como adultos
" is one of the following answers ("2 adultos y 1 niño") AND Question "." is one of the following answers ("No lo sé (no leer)","NC (no leer)"))

ID 276

135. Indique, por favor, si de media sus ingresos mensuales son más altos o más bajos que la cifra que aparece a continuación:
1668,40€ *

- Ingresan, al mes, más que esta cantidad
- Ingresan, al mes, aproximadamente esta cantidad (+-100€)
- Ingresan, al mes, menos que esta cantidad
- NS (no leer)
- NC (no leer)

LOGIC Hidden unless: (#88 Question "Por lo tanto, en su hogar conviven (contandose a usted):
* hace referencia a la última pregunta, de personas con las que convive y comparte gastos
* hijos de 14 años o más cuentan como adultos
" is one of the following answers ("2 adultos y 2 niños") AND Question "." is one of the following answers ("No lo sé (no leer)","NC (no leer)"))

ID 277

136. Indique, por favor, si de media sus ingresos mensuales son más altos o más bajos que la cifra que aparece a continuación: **1946,40€** *

- Ingresan, al mes, más que esta cantidad
- Ingresan, al mes, aproximadamente esta cantidad (+-100€)
- Ingresan, al mes, menos que esta cantidad
- NS (no leer)
- NC (no leer)

LOGIC Hidden unless: (#88 Question "Por lo tanto, en su hogar conviven (contandose a usted):
* hace referencia a la última pregunta, de personas con las que convive y comparte gastos
* hijos de 14 años o más cuentan como adultos
" is one of the following answers ("2 adultos y 3 niños") AND Question "." is one of the following answers ("No lo sé (no leer)","NC (no leer)"))

ID 278

137. Indique, por favor, si de media sus ingresos mensuales son más altos o más bajos que la cifra que aparece a continuación: **2224,50€** *

- Ingresan, al mes, más que esta cantidad
- Ingresan, al mes, aproximadamente esta cantidad (+-100€)
- Ingresan, al mes, menos que esta cantidad
- NS (no leer)
- NC (no leer)

LOGIC Hidden unless: (#88 Question "Por lo tanto, en su hogar conviven (contandose a usted):
* hace referencia a la última pregunta, de personas con las que convive y comparte gastos
* hijos de 14 años o más cuentan como adultos
" is one of the following answers ("3 adultos y 1 niño") AND Question "." is one of the following answers ("No lo sé (no leer)","NC (no leer)"))

ID 279

138. Indique, por favor, si de media sus ingresos mensuales son más altos o más bajos que la cifra que aparece a continuación: **2131, 80€** *

- Ingresan, al mes, más que esta cantidad
- Ingresan, al mes, aproximadamente esta cantidad (+-100€)
- Ingresan, al mes, menos que esta cantidad
- NS (no leer)
- NC (no leer)

LOGIC Hidden unless: (#88 Question "Por lo tanto, en su hogar conviven (contandose a usted):
* hace referencia a la última pregunta, de personas con las que convive y comparte gastos
* hijos de 14 años o más cuentan como adultos
" is one of the following answers ("3 adultos y 2 niños") AND Question " . " is one of the following answers ("No lo sé (no leer)","NC (no leer)"))

ID 280

139. Indique, por favor, si de media sus ingresos mensuales son más altos o más bajos que la cifra que aparece a continuación: **2409,90 €** *

- Ingresan, al mes, más que esta cantidad
- Ingresan, al mes, aproximadamente esta cantidad (+-100€)
- Ingresan, al mes, menos que esta cantidad
- NS (no leer)
- NC (no leer)

LOGIC Hidden unless: (#88 Question "Por lo tanto, en su hogar conviven (contandose a usted):
* hace referencia a la última pregunta, de personas con las que convive y comparte gastos
* hijos de 14 años o más cuentan como adultos
" is one of the following answers ("3 adultos y 3 niños") AND Question " . " is one of the following answers ("No lo sé (no leer)","NC (no leer)"))

ID 282

140. Indique, por favor, si de media sus ingresos mensuales son más altos o más bajos que la cifra que aparece a continuación: **2687,90 €** *

- Ingresan, al mes, más que esta cantidad
- Ingresan, al mes, aproximadamente esta cantidad (+-100€)
- Ingresan, al mes, menos que esta cantidad
- NS (no leer)
- NC (no leer)

LOGIC Hidden unless: (#88 Question "Por lo tanto, en su hogar conviven (contandose a usted):
* hace referencia a la última pregunta, de personas con las que convive y comparte gastos
* hijos de 14 años o más cuentan como adultos
" is one of the following answers ("4 adultos y 1 niño") AND Question " . " is one of the following answers ("No lo sé (no leer)","NC (no leer)"))

ID 283

141. Indique, por favor, si de media sus ingresos mensuales son más altos o más bajos que la cifra que aparece a continuación: **2595,20 €** *

- Ingresan, al mes, más que esta cantidad
- Ingresan, al mes, aproximadamente esta cantidad (+-100€)
- Ingresan, al mes, menos que esta cantidad
- NS (no leer)
- NC (no leer)

LOGIC Hidden unless: (#88 Question "Por lo tanto, en su hogar conviven (contandose a usted):
* hace referencia a la última pregunta, de personas con las que convive y comparte gastos
* hijos de 14 años o más cuentan como adultos
" is one of the following answers ("4 adultos y 2 niños") AND Question " . " is one of the following answers ("No lo sé (no leer)","NC (no leer)"))

ID 284

142. Indique, por favor, si de media sus ingresos mensuales son más altos o más bajos que la cifra que aparece a continuación: **2873,30 €** *

- Ingresan, al mes, más que esta cantidad
- Ingresan, al mes, aproximadamente esta cantidad (+-100€)
- Ingresan, al mes, menos que esta cantidad
- NS (no leer)
- NC (no leer)

LOGIC Hidden unless: (#88 Question "Por lo tanto, en su hogar conviven (contandose a usted):
* hace referencia a la última pregunta, de personas con las que convive y comparte gastos
* hijos de 14 años o más cuentan como adultos
" is one of the following answers ("4 adultos y 3 niños") AND Question "." is one of the following answers ("No lo sé (no leer)","NC (no leer)"))

ID 285

143. Indique, por favor, si de media sus ingresos mensuales son más altos o más bajos que la cifra que aparece a continuación: **3151,30 €** *

- Ingresan, al mes, más que esta cantidad
- Ingresan, al mes, aproximadamente esta cantidad (+-100€)
- Ingresan, al mes, menos que esta cantidad
- NS (no leer)
- NC (no leer)

LOGIC Hidden unless: (#88 Question "Por lo tanto, en su hogar conviven (contandose a usted):
* hace referencia a la última pregunta, de personas con las que convive y comparte gastos
* hijos de 14 años o más cuentan como adultos
" is one of the following answers ("5 adultos y 1 niño") AND Question "." is one of the following answers ("No lo sé (no leer)","NC (no leer)"))

ID 286

144. Indique, por favor, si de media sus ingresos mensuales son más altos o más bajos que la cifra que aparece a continuación: **3058,70 €** *

- Ingresan, al mes, más que esta cantidad
- Ingresan, al mes, aproximadamente esta cantidad (+-100€)
- Ingresan, al mes, menos que esta cantidad
- NS (no leer)
- NC (no leer)

LOGIC Hidden unless: (#88 Question "Por lo tanto, en su hogar conviven (contandose a usted):
* hace referencia a la última pregunta, de personas con las que convive y comparte gastos
* hijos de 14 años o más cuentan como adultos
" is one of the following answers ("5 adultos y 2 niños") AND Question "." is one of the following answers ("No lo sé (no leer)","NC (no leer)"))

ID 287

145. Indique, por favor, si de media sus ingresos mensuales son más altos o más bajos que la cifra que aparece a continuación: **3336,70 €** *

- Ingresan, al mes, más que esta cantidad
- Ingresan, al mes, aproximadamente esta cantidad (+-100€)
- Ingresan, al mes, menos que esta cantidad
- NS (no leer)
- NC (no leer)

LOGIC Hidden unless: (#88 Question "Por lo tanto, en su hogar conviven (contandose a usted):
* hace referencia a la última pregunta, de personas con las que convive y comparte gastos
* hijos de 14 años o más cuentan como adultos
" is one of the following answers ("5 adultos y 3 niños") AND Question "." is one of the following answers ("No lo sé (no leer)","NC (no leer)"))

ID 288

146. Indique, por favor, si de media sus ingresos mensuales son más altos o más bajos que la cifra que aparece a continuación: **3614,80 €** *

- Ingresan, al mes, más que esta cantidad
- Ingresan, al mes, aproximadamente esta cantidad (+-100€)
- Ingresan, al mes, menos que esta cantidad
- NS (no leer)
- NC (no leer)

LOGIC Hidden unless: (#88 Question "Por lo tanto, en su hogar conviven (contandose a usted):
* hace referencia a la última pregunta, de personas con las que convive y comparte gastos
* hijos de 14 años o más cuentan como adultos
" is one of the following answers ("Otros") AND Question "." is one of the following answers ("No lo sé (no leer)","NC (no leer)"))

ID 522

147. Indique, por favor, si de media sus ingresos mensuales son más altos o más bajos que la cifra que aparece a continuación: (ver tabla excel según composición del hogar) *

- Ingresan, al mes, más de esta cantidad
- Ingresan, al mes, aproximadamente esta cantidad (+-100€)
- Ingresan, al mes, menos de esta cantidad
- NS (no leer)
- NC (no leer)

ID 301

148. En su hogar, tiene los equipamientos siguientes i pueden hacer uso? *

	Sí	No	NS (no leer)	NC (no leer)
Lavadora				
Televisor en color				
Teléfono (fijo o móvil)				
Ordenador (portátil o sobremesa)				
Automóvil (se incluye el coche de empresa disponible para uso personal)				
Acceso a internet				

LOGIC Hidden unless: Question "Lavadora" is one of the following answers ("No")
ID 374
149. La lavadora, se la pueden permitir?
- Sí
- No

LOGIC Hidden unless: Question "Televisor en color" is one of the following answers ("No")
ID 375
150. La televisión, se la pueden permitir?
- Sí
- No

LOGIC Hidden unless: Question "Teléfono (fijo o móvil)" is one of the following answers ("No")
ID 376
151. El teléfono, se lo pueden permitir?
- Sí
- No

LOGIC Hidden unless: Question "Ordenador (portátil o sobremesa)" is one of the following answers ("No")
ID 377
152. El ordenador, se lo pueden permitir?
- Sí
- No

LOGIC Hidden unless: Question "Automóvil (se incluye el coche de empresa disponible para uso personal)" is one of the following answers ("No")

ID 378

153. El coche, se lo pueden permitir?

- Sí
- No

LOGIC Hidden unless: Question "Acceso a internet" is one of the following answers ("No")

ID 379

154. El acceso a internet, se lo pueden permitir?

- Sí
- No

ID 302

155. Me podría decir si su hogar se pueden permitir.... *

	Sí	No	NS (no leer)	NC (no leer)
Ir de vacaciones al menos una semana al año?				
Una comida de carne, pollo o pescado (o equivalente para vegetarianos) al menos cada dos días?				
Hacer frente a un gasto imprevisto de 927€ con sus propios recursos?				
Realizar actividades de ocio como ir al cine, conciertos, comer fuera... al menos una vez al mes?				
Mantener su hogar con una temperatura adecuada durante los meses fríos				

ID 306

156. Me podría indicar si en los últimos 12 meses su hogar se ha atrasado en el pago de un recibo o cuota de los gastos siguientes, debido a dificultades económicas? *

	No tiene que realizar este tipo de pago	Sí, solo una vez	Sí, dos veces o más	No	NS (no leer)	NC (no leer)
Préstamos hipotecarios de la vivienda habitual o recibo del alquiler	○	○	○	○	○	○
Compras aplazadas o otros préstamos (préstamos hipotecarios de segundas residencias, pero no del hogar principal)	○	○	○	○	○	○
Facturas o recibos de servicios (luz, agua, gas calefacción, comunidad...)	○	○	○	○	○	○

ID 313

157. Considerando el total de ingresos netos mensuales de su hogar, como acostumbra a llegar a final de mes? *

- ○ Con mucha dificultad
- ○ Con dificultad
- ○ Con cierta dificultad
- ○ Con cierta facilidad
- ○ Con facilidad
- ○ Con mucha facilidad
- ○ NS (no leer)
- ○ NC (no leer)

ID 391

158. A qué edad empezó a trabajar de forma remunerada como actividad principal?

- Años
- NS (no leer)
- NC (no leer)

LOGIC Show/hide trigger exists.

ID 317

159. Durante los últimos 10 años, ha estado alguna vez en situación de desempleo (queriendo trabajar)? *

- No
- Sí
- NS (no llegir)
- NC (no llegir)

LOGIC Hidden unless: #159 Question "Durante los últimos 10 años, ha estado alguna vez en situación de desempleo (queriendo trabajar)?" is one of the following answers ("Sí")

ID 318

160. Durante cuanto tiempo aproximadamente ha estado en desempleo en total en los últimos 10 años?
*

- Menos de 1 año
- Entre 1 y 2 años (2 no incluido)
- Entre más de 2 y 4 años (4 no incluido)
- 4 años o más
- NS (no lee)
- NC (no leer)

LOGIC Hidden unless: #159 Question "Durante los últimos 10 años, ha estado alguna vez en situación de desempleo (queriendo trabajar)?" is one of the following answers ("Sí")

ID 320

161. Respecto a la última vez que estuvo en situación de desempleo, cuanto tiempo estuvo en esa situación?

- Años
- Meses
- NS (no leer)
- NC (no leer)

ID 321

162. En los últimos tres años, cuantos contratos ha tenido? *

- Ninguno
- El vigente actual
- Dos contratos
- De 3 a 5
- De 6 a 10
- D'11 a 20
- Más de 20
- NS (no leer)
- NC (no leer)

322
163. En relación a los trabajos realizados en los últimos 3 años, alguno de ellos ha sido con contrato indefinido? *

- No
- Si
- NS (no leer)
- NC (no leer)

323
164. En relación a los trabajos realizados en los últimos 3 años, algun de ellos ha sido sin contrato? *

- No
- Si
- NS (no leer)
- NC (no leer)

Varios

Page description:

324
165. Realiza alguna actividad para reducir o prevenir el estrés? *

- No
- NS(no leer)
- NC (no leer)

Podría mencionar cuál?

- Sí

LOGIC Show/hide trigger exists.

ID 328

166. Para terminar, nos gustaría solicitarle si le importaría que le volviéramos a contactar dentro de unos meses para profundizar en algunas partes de este estudio, en una segunda fase de la investigación. **En todo momento podrá decir que no quiere participar.**

En el caso que diga que sí, darle la hoja de consentimiento de recontacto. *

- ○ No

Teléfono

- ○ Si

LOGIC Hidden unless: #166 Question "Para terminar, nos gustaría solicitarle si le importaría que le volviéramos a contactar dentro de unos meses para profundizar en algunas partes de este estudio, en una segunda fase de la investigación. **En todo momento podrá decir que no quiere participar.** En el caso que diga que sí, darle la hoja de consentimiento de recontacto." is one of the following answers ("Si")

ID 380

167. Teléfono

[ ]

LOGIC Hidden unless: #166 Question "Para terminar, nos gustaría solicitarle si le importaría que le volviéramos a contactar dentro de unos meses para profundizar en algunas partes de este estudio, en una segunda fase de la investigación. **En todo momento podrá decir que no quiere participar.** En el caso que diga que sí, darle la hoja de consentimiento de recontacto." is one of the following answers ("Si")

ID 381

168. E-mail

[ ]

LOGIC Show/hide trigger exists.

ID 386

169. Estaría interesado en recibir los resultados de la analítica sobre la concentración de cortisol en su pelo? *

- Sí
- No

LOGIC Hidden unless: #169 Question "Estaría interesado en recibir los resultados de la analítica sobre la concentración de cortisol en su pelo?" is one of the following answers ("Sí")

ID 388

170. E-mail

Si ya han puesto el email a la pregunta anterior, no hace falta rellenarlo de nuevo

ID 329

171. Observaciones

ID 330

172. Incidencias

VALIDATION Must be numeric

ID 331

173. Hora de finalización *
